# Natural polysaccharide-engineered POP@ZIF-67 nanoparticles coordinate antibacterial activity, macrophage polarization, and osteogenesis for chronic osteomyelitis therapy

**DOI:** 10.3389/fbioe.2026.1935416

**Published:** 2026-10-07

**Authors:** Yunchang Zhao, Guangting Zhu, Mengxue Zhang, Yize Wu, Junwei Su, Jing Hu, Chao Jian, Ying Yuan

**Affiliations:** 1 Department of Orthopedics Trauma and Microsurgery, Zhongnan Hospital of Wuhan University, Wuhan, China; 2 Hubei Provincial Clinical Medical Research Center for Trauma and Microsurgery, Wuhan, China

**Keywords:** bone regeneration, chronic osteomyelitis, macrophage polarization, osteoimmunomodulation, Portulaca oleracea polysaccharide, zeolitic imidazolate framework-67

## Abstract

Chronic osteomyelitis is difficult to treat due to the interplay of bacterial colonization, excessive inflammation, and impaired bone regeneration. Here, we developed an engineered natural polysaccharide POP@ZIF-67 nanoplatform to modulate these interrelated pathological processes. ZIF-67 is an antimicrobial framework with porous properties, peroxidase-like catalytic activity, and acid-responsive release behavior; purslane polysaccharide (POP) was incorporated as an immunomodulatory component to mitigate the excessive inflammatory stimulation induced by ZIF-67. POP@ZIF-67 exhibits effective antibacterial and antibiofilm activity against *Staphylococcus aureus*; more importantly, the incorporation of POP transforms the biological action of ZIF-67 from primarily antibacterial stimulation into a multifunctional platform capable of suppressing excessive M1-like macrophage responses, promoting repair-related polarization, and improving the osteogenic microenvironment. In a mouse model of chronic osteomyelitis, POP@ZIF-67 reduced local bacterial load and inflammatory damage while promoting collagen deposition, bone remodeling, and osteogenic maturation, without significant systemic toxicity. Overall, the study introduces an “antimicrobial framework–immune buffering” strategy that reconciles the antimicrobial efficacy of metal-organic frameworks with their immunostimulatory limitations. By integrating various therapeutic approaches, POP@ZIF-67 provides a promising new synthetic paradigm for the treatment of chronic osteomyelitis and infectious bone defects.

## 1 Introduction

Chronic osteomyelitis is a refractory bone disease caused by persistent infection with pathogenic bacteria. *Staphylococcus aureus (Staphylococcus aureus)* is one of the most significant pathogens in osteomyelitis; through mechanisms such as biofilm formation and immune evasion, it limits the effectiveness of both antibiotic therapy and surgical debridement. Therefore, chronic osteomyelitis is not merely a bacterial infection but a complex pathological process driven by bacterial colonization, biofilm formation, immune imbalance, and impaired bone regeneration (Song et al., 2025; Brandquist and Kielian, 2025).

Macrophages are key cells in both the antibacterial immune response and tissue repair; their phenotypic changes directly influence infection and tissue regeneration. A moderate M1-like response aids in pathogen clearance, but sustained M1-like polarization hinders the tissue repair process; M2-like macrophages, on the other hand, help promote anti-inflammatory responses, matrix remodeling, and bone tissue repair. Therefore, the design of therapeutic materials for chronic osteomyelitis should shift from a single antimicrobial strategy toward comprehensive therapeutic platforms that simultaneously possess antimicrobial, immunomodulatory, and osteogenic capabilities (Geng et al., 2025; Wang et al., 2025).

Metal–organic framework (MOF) materials have attracted the attention of researchers in anti-infective therapy and bone tissue engineering due to their high specific surface area, tunable pore structure, metal ion nodes, and stimulus-responsive release properties. Among these, zeolitic imidazolate framework-67 (ZIF-67) can inhibit bacterial growth through metal ion release, ROS regulation, and local catalytic reactions, making it suitable as an antimicrobial framework and delivery platform for the treatment of bone infections. However, relying solely on the antimicrobial effects of ZIF-67 still has certain limitations. While these functions kill bacteria, they can also adversely affect macrophages and induce excessive pro-inflammatory responses, which may further damage the post-infectious osteogenic microenvironment. Therefore, the key challenge in enhancing the application value of ZIF-67 in the treatment of chronic osteomyelitis is how to neutralize its potential pro-inflammatory tendencies while retaining its advantages as a carrier and its antimicrobial efficacy.

Natural polysaccharides possess good biocompatibility and immunomodulatory activity, making them important components for constructing mild immunomodulatory materials. *Portulaca oleracea polysaccharide* (POP) has been reported to have the potential to modulate the inflammatory response of macrophages and may be associated with inflammation-related pathways such as ERK/NF-κB (He et al., 2023; Wang et al., 2026). Furthermore, some plant-derived polysaccharides can enhance osteoblast activity and promote osteogenic differentiation, suggesting that natural polysaccharides have certain application value in regulating the bone repair microenvironment. Therefore, in this study, POP was incorporated into the ZIF-67 system to serve as an immunomodulatory component, aiming to mitigate the pro-inflammatory tendencies that ZIF-67 may induce while improving the osteogenic microenvironment under infection stress.

Although a large number of nanomaterials have been used in research on the treatment of chronic osteomyelitis, most MOF-based materials still primarily emphasize antibacterial effects, with insufficient attention paid to infection-related immune imbalances and impaired bone repair. Furthermore, the oxidative stress and pro-inflammatory effects associated with some MOF materials may impair tissue regeneration in the complex microenvironment of infections. In contrast, the POP@ZIF-67 material designed in this study serves as a multifunctional integrated platform: ZIF-67 endows the material with antibacterial, antibiofilm, peroxidase-like catalytic activity, and acid-responsive release properties, while POP acts as an immunomodulatory component, buffering excessive inflammatory responses and promoting the remodeling of the osteogenic microenvironment. This complementary design distinguishes POP@ZIF-67 from previously reported MOF-based materials that primarily exert antibacterial or single-function immunomodulatory effects.

We hypothesize that incorporating POP into ZIF-67 will preserve the antimicrobial efficacy of ZIF-67 while attenuating its potential pro-inflammatory effects. Therefore, POP@ZIF-67 is expected to promote the transition of macrophages toward a reparative phenotype, reduce infection-related inflammation and bacterial load, and facilitate bone regeneration in chronic osteomyelitis. To validate this hypothesis, this study systematically evaluated the physicochemical properties of POP@ZIF-67, its antibacterial and antibiofilm activities, its effects on macrophage polarization and cytokine secretion, its osteogenic activity under infection-related stress conditions, and its therapeutic efficacy in a mouse model of chronic osteomyelitis.

## 2 Results

### 2.1 Preparation and characterization of POP@ZIF-67

ZIF-67 was prepared via the self-assembly of Co^2+^ and 2-methylimidazole through coordination, and POP was subsequently introduced to construct the POP@ZIF-67 composite system. The resulting ZIF-67 dispersion exhibited a characteristic blue-violet colour, indicating the successful formation of the cobalt-based ZIF-67 framework (Figure 1a). As a metal-organic framework formed by the coordination of Co^2+^ with 2-methylimidazole, ZIF-67 possesses a high specific surface area, a regular pore structure and favourable carrier properties, and has been utilised in drug/enzyme loading and the design of biomedical materials (Zhang et al., 2025). Subsequently, to preliminarily evaluate the peroxidase-like activity of ZIF-67, the catalytic effect of different concentrations of ZIF-67 on the TMB/H_2_O_2_ colourimetric system was examined at pH 6.5. As the ZIF-67 concentration increased from 10 μg/mL to 160 μg/mL, the colour of the reaction system gradually deepened, indicating that ZIF-67 possesses concentration-dependent POD-like catalytic activity (Figure 1b). These findings confirm the concentration-dependent POD-like catalytic activity of ZIF-67 under the tested conditions. However, they do not establish whether this activity directly contributes to the antibacterial effect of POP@ZIF-67. Similarly, ZIF-67 and its composite nanomaterials have been employed in TMB/H_2_O_2_ colourimetric reactions and the evaluation of POD-like nanozymes (Cui et al., 2025; Zhu et al., 2022). Furthermore, the enzymatic-like activity of MOF-based nanozymes can be modulated via metal sites, pore confinement and framework structure, which also provides a theoretical basis for the catalytic application of ZIF-67 in antimicrobial microenvironments (Yu et al., 2025).

**FIGURE 1:**
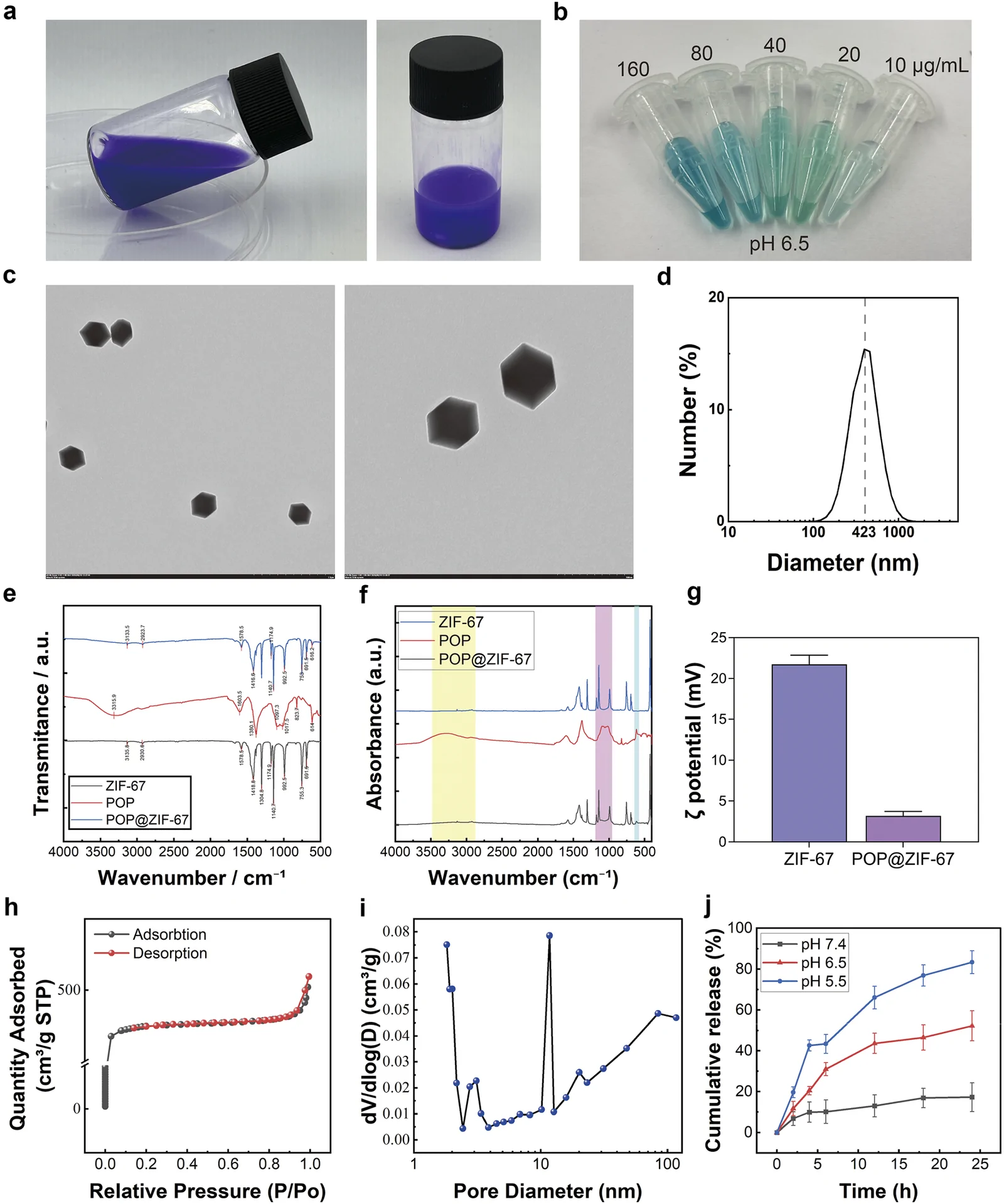
Preparation and characterization of POP@ZIF-67. **(a)** Macroscopic photograph of ZIF-67 dispersion. **(b)** Concentration-dependent peroxidase-like activity of ZIF-67 in a TMB/H_2_O_2_ system. **(c)** TEM image of ZIF-67 nanoparticles. **(d)** DLS particle size distribution of ZIF-67. **(e)** FT-IR transmittance spectra of ZIF-67, POP and POP@ZIF-67. **(f)** FT-IR absorbance spectra of ZIF-67, POP and POP@ZIF-67. **(g)** ζ-potential of ZIF-67 and POP@ZIF-67. **(h)** N_2_ adsorption–desorption isotherms of POP@ZIF-67. **(i)** Pore size distribution curves of POP@ZIF-67. **(j)** Cumulative POP release curves of POP@ZIF-67 at pH 7.4, pH 6.5 and pH 5.5. Data are expressed as mean ± SD, n = 3.

TEM results show that ZIF-67 exhibits a regular polyhedral morphology with good particle dispersion, suggesting that the material possesses the characteristic crystal structure of ZIF-67 (Figure 1c). DLS particle size analysis indicates that the hydrated particle size is primarily distributed around 423 nm (Figure 1d). The DLS particle size is slightly larger than that observed by TEM, which may be attributed to the hydration layer, slight agglomeration and differences in testing conditions. In studies of ZIF-type nanoparticles, TEM is typically used to observe dry-state morphology, whilst DLS is employed to analyse hydrated particle size and dispersion state; therefore, a certain degree of discrepancy between the two is reasonable (Zhang et al., 2025; Yu et al., 2026).

To verify the composite formation between POP and ZIF-67, further FTIR analysis was conducted. The ZIF-67 group exhibited characteristic peaks associated with imidazole ring skeletal vibrations and Co–N coordination at 1,500–1,600 cm^-1^, 1,300–1,400 cm^-1^, and in the low-wavenumber region. The POP group, on the other hand, exhibited absorption features typical of polysaccharides, including–OH stretching vibrations near 3,200–3,400 cm^-1^, C–H vibrations near 2,900 cm^-1^, and C–O–C/C–O vibrations in the 1,000–1,100 cm^-1^ region. POP@ZIF-67 retained both the ZIF-67 framework peaks and the characteristic polysaccharide peaks of POP, accompanied by partial changes in peak intensity or slight shifts in peak positions, indicating that POP had been successfully incorporated into the ZIF-67 system (Figures 1e,f; Supplementary Figure S1). Studies on SCG/ZIF-67/GOX and LA@ZIF-8, amongst others, have also frequently demonstrated the successful formation of composite materials through the coexistence of characteristic peaks of the guest molecules and the ZIF framework peaks (Zhang et al., 2025; Zhang et al., 2025).

The ζ-potential further confirms the modification of the ZIF-67 surface by POP. The surface potential of ZIF-67 is approximately +22 mV, whereas that of POP@ZIF-67 is reduced to approximately +3 mV (Figure 1g). This change may stem from the shielding of the positive charges on the ZIF-67 surface by hydrophilic/anionic groups such as hydroxyl and carboxyl groups in POP, and also indicates that POP has coated or adsorbed onto the ZIF-67 surface. Similarly, following the modification of ZIF-8 with LA, the surface potential of the material shifted from positive to negative, which was used to demonstrate the successful introduction of hydroxyl- and phenolic hydroxyl-rich molecules (Zhang et al., 2025).

BET analysis showed that ZIF-67 possessed a high specific surface area of 1,490.97 m^2^/g (Supplementary Figure S2), confirming the highly porous nature of the framework and providing a favorable structural basis for POP loading. The N_2_ adsorption–desorption curves show that the material exhibits significant adsorption in the low relative pressure region and an increase in adsorption capacity in the high relative pressure region, suggesting that it possesses a typical MOF porous structure (Figure 1h). Pore size distribution results further indicate the presence of a microporous/mesoporous structure (Figure 1i), which provides the spatial basis for the loading and subsequent release of POP. Existing studies on ZIF-8 and ZIF-67 have shown that the porous structure, high specific surface area and tunable pore characteristics of ZIF-type MOFs form an important basis for their use as drug delivery platforms and carriers for functional materials (Zhang et al., 2025; Yu et al., 2026; Sun et al., 2026; Tran et al., 2025).

To further verify the crystal structure and elemental composition of the POP@ZIF-67 composite, we conducted additional XRD and XPS analyses. As shown in Supplementary Figure S3, POP@ZIF-67 retained the characteristic diffraction peaks of standard ZIF-67, indicating that the introduction of POP did not disrupt the crystal framework of ZIF-67. Furthermore, XPS analysis confirmed the presence of cobalt in the composite; the characteristic Co 2p_1_/_2_ and Co 2p_3_/_2_ peaks confirmed the retention of cobalt species within the POP@ZIF-67 composite system (Supplementary Figure S4).

Given that the biological function of ZIF-67 is closely related to cobalt ion release, we further investigated the Co^2+^ release behavior of POP@ZIF-67. As shown in Supplementary Figure S5, POP@ZIF-67 exhibited a distinct time-dependent cobalt ion release process, with rapid release in the early stages, followed by a gradual decrease in the release rate until reaching a relatively stable plateau, with continuous Co^2+^ release over 96 h. These results indicate that POP@ZIF-67 exhibits sustained cobalt ion release behavior, providing a potential basis for its subsequent antibacterial activity.

Finally, the release behaviour of POP@ZIF-67 under different pH conditions was investigated. The results show that POP release is relatively slow at pH 7.4; release increases significantly at pH 6.5; and release is fastest at pH 5.5, with a cumulative release rate of over 80% after 24 h (Figure 1j). This pH-dependent release behaviour indicates that POP@ZIF-67 exhibits a certain degree of stability under neutral conditions, whilst the release of POP is accelerated in infection- or inflammation-associated acidic microenvironments. The acid sensitivity of ZIF-67 is typically associated with the gradual disruption of the Co–N coordination structure under acidic conditions; this characteristic has been utilised in acid-responsive release systems involving ZIF-67 loaded with GOX (Zhang et al., 2025). Similarly, ZIF-8 systems often exhibit accelerated release under acidic conditions, which can be used to explain the responsive delivery behaviour of ZIF-type materials in the acidic microenvironments associated with inflammation or infection (Zhang et al., 2025; Yu et al., 2026; Tran et al., 2025).

In summary, POP@ZIF-67 simultaneously exhibits the regular crystal morphology of ZIF-67, POP-related functional group characteristics, changes in surface potential, a porous structure, and acid-responsive release behaviour, demonstrating that POP has been successfully integrated into the ZIF-67 system. This composite material serves as a pH-responsive delivery platform for POP and retains the POD-like activity and porous carrier characteristics of ZIF-67. The retained POD-like activity may represent a possible antibacterial mechanism.

### 2.2 *In vitro* antimicrobial and antibiofilm properties of POP@ZIF-67

The persistent progression of chronic osteomyelitis is closely associated with residual bacteria and biofilm formation; therefore, evaluating the inhibitory effects of POP@ZIF-67 is a critical step in verifying its anti-infective potential. MOF-based antimicrobial materials may exert their antibacterial effects through multiple mechanisms, including metal ion release, disruption of bacterial cell walls, reactive oxygen species (ROS) generation, and drug delivery (Gallegos-Monterrosa et al., 2023; Chen et al., 2024).

Quantitative analysis of the plate-count results showed that the bacterial loads were approximately 8.5, 8.0, 6.8, and 6.1 log_10_(CFU/mL) in the Control, POP, ZIF-67, and POP@ZIF-67 groups, respectively (Figures 2A,D). Compared with the Control group, bacterial loads were significantly reduced in the ZIF-67 and POP@ZIF-67 groups (P < 0.0001), with POP@ZIF-67 showing a lower bacterial load than ZIF-67. Consistent with these findings, the relative bacterial viability was approximately 100%, 84%, 22%, and 13% in the four groups, respectively (Figures 2B,E). Biofilm thickness was approximately 4.4, 3.8, 1.9, and 1.7 μm in the Control, POP, ZIF-67, and POP@ZIF-67 groups, respectively (Figures 2C,F). These results demonstrate that POP@ZIF-67 exerts stronger antibacterial and antibiofilm effects than POP or ZIF-67 alone.

**FIGURE 2:**
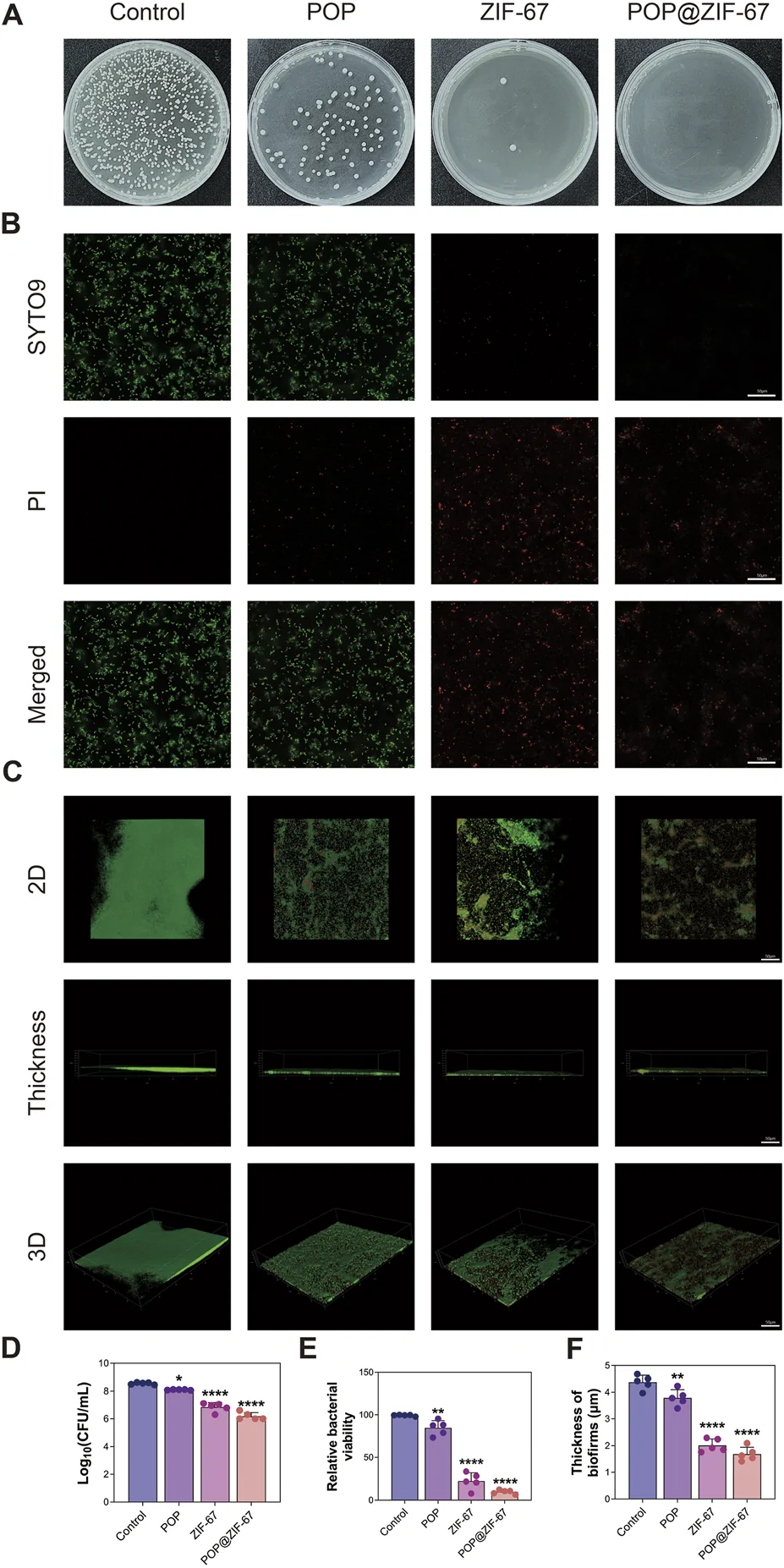
Antibacterial and antibiofilm activities of POP@ZIF-67. **(A)** Image of a bacterial plate spread. **(B)** Representative image of bacterial live/dead staining results; scale bar = 50 μm. **(C)** Bacterial biofilm structure; scale bar = 50 μm. **(D)** Quantitative analysis of bacterial colony counts. **(E)** Quantitative analysis of relative bacterial survival rates. **(F)** Quantitative analysis of biofilm thickness. Data are expressed as mean ± SD, n = 5; *p < 0.05; **p < 0.01; ****p < 0.0001.

SYTO9/PI live/dead staining was used to further observe changes in bacterial viability after different treatments. The Control and POP groups exhibited predominantly green fluorescence with minimal red PI fluorescence, indicating that most bacteria were still alive. After ZIF-67 treatment, green fluorescence decreased significantly, while the red signal from dead bacteria increased; in the POP@ZIF-67 group, green fluorescence decreased further, suggesting the strongest inhibition of bacterial activity (Figure 2B). Corresponding quantitative results for relative bacterial activity also showed the POP group exhibited only a slight decrease in bacterial activity, whereas the ZIF-67 and POP@ZIF-67 groups exhibited a significant decline in bacterial survival rate (Figure 2E). This result is consistent with the plate spread experiment and further demonstrates that POP@ZIF-67 possesses a distinct bactericidal effect.

Confocal laser scanning microscopy (CLSM) was further employed to examine the effects of each group on bacterial biofilm formation. The Control group formed a continuous and dense biofilm structure, with a large area of green fluorescence in the 2D images; both the thickness map and 3D reconstruction showed a relatively thick biofilm layer. Biofilm thickness decreased after POP treatment, suggesting that POP may exert a certain inhibitory effect on bacterial adhesion or biofilm formation. In the ZIF-67 group, the continuity of the biofilm was markedly disrupted, and the biofilm thickness was significantly reduced. The POP@ZIF-67 group exhibited the thinnest biofilm, and the 3D reconstruction revealed the least residual biofilm structure (Figure 2C). Quantitative results showed that POP, ZIF-67, and POP@ZIF-67 all reduced biofilm thickness to varying degrees, with the POP@ZIF-67 group exhibiting the most pronounced inhibitory effect (Figure 2F).

In summary, POP@ZIF-67 exhibited antibacterial and antibiofilm activities under the tested conditions. The specific contributions of Co^2+^ release and POD-like ROS generation require further investigation.

### 2.3 POP@ZIF-67 promotes osteogenic differentiation under infection-induced stress conditions

Previous studies have shown that materials with both antibacterial and osteogenic properties are more suitable for the repair of infected bone defects (Zhang et al., 2023; Jia et al., 2022). We further evaluated the ability of POP@ZIF-67 to improve the osteogenic microenvironment under infection-induced stress conditions. In this section of the experiment, the Control group consisted of MC3T3-E1 cells co-cultured with inactivated *S. aureus* to simulate the cellular stress state associated with bone infection. The proliferation activity of MC3T3-E1 cells treated with different materials was assessed using the CCK-8 assay. The results showed that, under the stress of co-culture with inactivated *S. aureus*, the cells in the Control group maintained a certain level of proliferation capacity as the culture time increased. The ZIF-67 group showed no significant differences compared to the Control group at any time point, indicating that ZIF-67 did not exhibit significant cytotoxicity toward MC3T3-E1 cells at the current treatment concentration (Figure 3A). Both the POP group and the POP@ZIF-67 group significantly increased cell viability at 48 and 72 h. These results indicate that POP is the primary component contributing to the promotion of MC3T3-E1 cell proliferation, and that ZIF-67 loading did not impair POP’s cell compatibility or proliferative effects.

**FIGURE 3:**
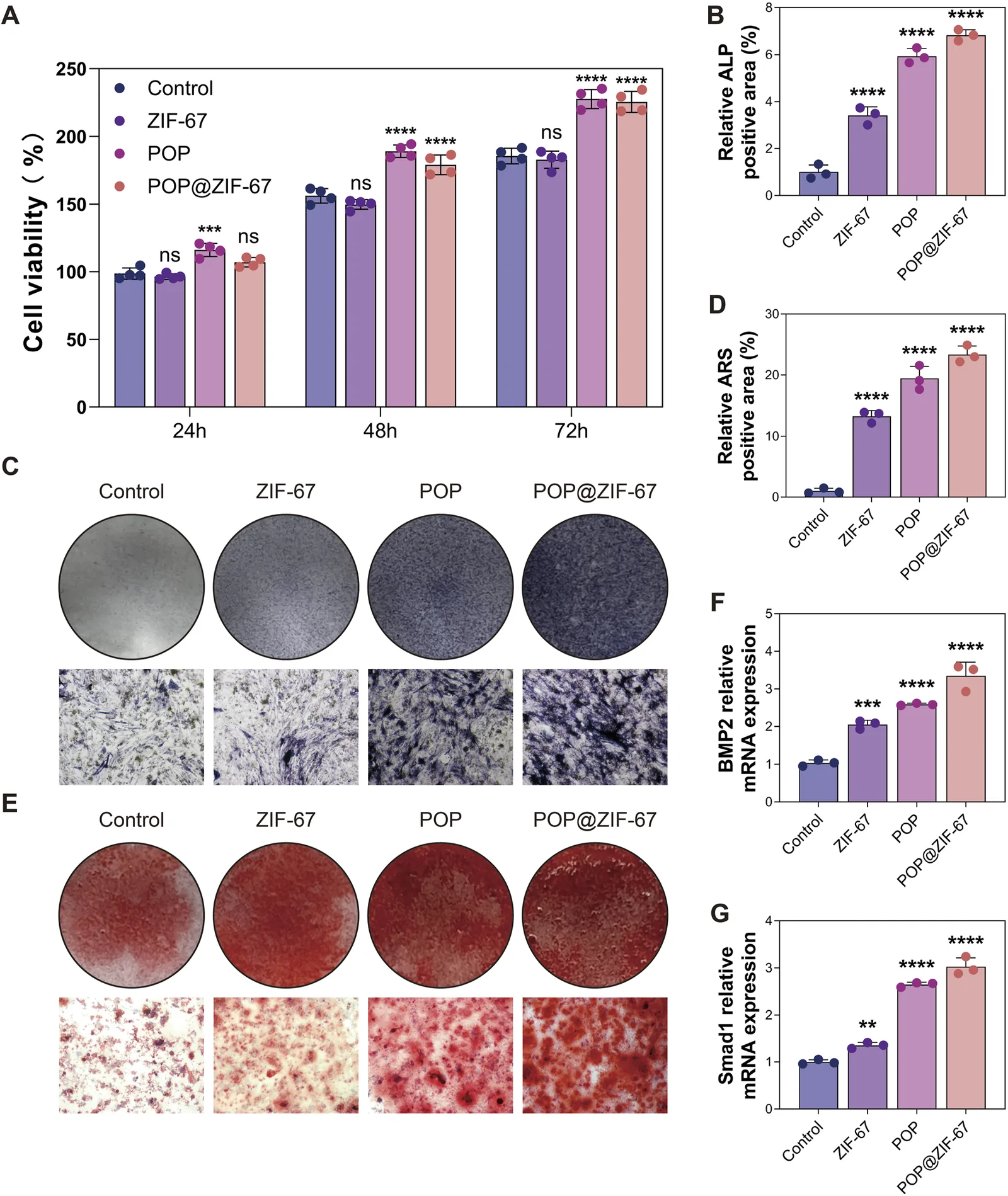
Effect of POP@ZIF-67 on osteogenic differentiation of MC3T3-E1 cells under infection stress conditions. **(A)** CCK-8 cell viability assay of MC3T3-E1 cells. **(B)** Quantitative analysis of ALP-positive area. **(C)** Representative image of ALP staining. **(D)** Quantitative analysis of ARS-positive area. **(E)** Representative image of ARS staining. **(F)** Relative expression levels of *BMP2* mRNA. **(G)** Relative expression levels of *Smad1* mRNA. Data are presented as mean ± SD, n = 3. ns, not significant; **p < 0.01, ***p < 0.001, ****p < 0.0001.

Early osteogenic differentiation was evaluated through ALP staining and quantitative analysis. Compared with the infection-stress control group, the ZIF-67 group exhibited an increase in ALP-positive area, suggesting that ZIF-67 may possess some osteogenic potential. Quantitative analysis showed that the relative ALP-positive areas were approximately 1.0%, 3.3%, 5.8%, and 6.8% in the Control, ZIF-67, POP, and POP@ZIF-67 groups, respectively (Figures 3B,C). (Sun et al., 2021) The POP@ZIF-67 group showed a significantly larger ALP-positive area than the Control group (*P* < 0.0001) and the ZIF-67 group (*P* < 0.0001). Similarly, the relative ARS-positive areas were approximately 0.7%, 13.0%, 19.5%, and 23.0% in the four groups, respectively (Figures 3D,E). These results indicate that POP@ZIF-67 enhances both early osteogenic differentiation and late-stage matrix mineralization under infection-related stress conditions (Zhang et al., 2023; Jia et al., 2022).

To further investigate the potential molecular basis of POP@ZIF-67’s promotion of osteogenic differentiation, the mRNA expression of *Bmp2* and *Smad1* was analyzed. The results showed that, compared with the infection-stress control group, ZIF-67 upregulated *Bmp2* and *Smad1* expression; the POP group exhibited a more pronounced upregulatory effect, while the POP@ZIF-67 group had the highest expression levels of Bmp2 and Smad1 (Figures 3F,G). The *BMP2/Smad* signaling pathway is a key regulator of osteogenic differentiation; its activation promotes *BMP2*-induced phosphorylation of *Smad1/5/8*, which in turn drives osteogenesis-related transcriptional programs (Zhang et al., 2024; Wang et al., 2023; Song et al., 2024).

To further verify whether the BMP/Smad signaling pathway is regulated at the protein level, we measured the levels of p-Smad1/5/9 and total Smad1/5/9 in MC3T3-E1 cells and performed densitometric analysis of the p-Smad1/5/9-to-total Smad1/5/9 ratio. The results showed that there was no significant difference in the p-Smad1/5/9-to-total Smad1/5/9 ratio between the Control group and the ZIF-67 group; compared with the Control group, this ratio was significantly elevated in the POP group, and it was also significantly elevated in the POP@ZIF-67 group compared with the ZIF-67 group. No significant difference was observed between the POP group and the POP@ZIF-67 group (Supplementary Figure S6). These results suggest that ZIF-67 treatment alone did not significantly promote Smad1/5/9 phosphorylation, whereas POP may be the primary functional component responsible for activating the BMP/Smad signaling pathway. POP retained its ability to promote Smad1/5/9 phosphorylation after being loaded onto ZIF-67. Therefore, the pro-osteogenic effects of POP and POP@ZIF-67 may be partially related to POP-mediated activation of the BMP/Smad signaling pathway.

In summary, under the conditions of infection stress caused by co-culture with inactivated *S. aureus*, POP@ZIF-67 maintained good cellular compatibility and significantly enhanced ALP activity, mineralized nodule formation, and the expression of *BMP2/Smad1*-related osteogenic genes in MC3T3-E1 cells. These results indicate that POP@ZIF-67 can improve osteogenic differentiation under infectious conditions.

### 2.4 POP@ZIF-67 regulates macrophage polarization under infection stress and enhances their antimicrobial clearance function

The treatment of chronic osteomyelitis also requires the regulation of excessive inflammatory responses in the infection microenvironment. Macrophages are key cells in the immune response to bone infections, performing functions such as phagocytosis and clearance of pathogens, regulation of inflammatory responses, and participation in tissue repair (Chen et al., 2023; Shi et al., 2025). We further evaluated the effects of POP@ZIF-67 on the activity, polarization, and antibacterial clearance function of RAW264.7 macrophages under infection stress conditions. The Control group consisted of RAW264.7 cells co-cultured with inactivated *S. aureus* to simulate an infection-related immune stimulation state. The biocompatibility of RAW264.7 cells treated with different materials was evaluated using live/dead staining. The results showed that all groups exhibited predominantly green signals for live cells and few red signals for dead cells, indicating that none of the treatments induced significant cell death at the current concentrations (Figure 4A). CCK-8 results further demonstrated that POP was the primary factor contributing to improved RAW264.7 cell viability and biocompatibility; although ZIF-67 did not cause significant cytotoxicity, it did not significantly promote macrophage proliferation or metabolic activity on its own (Figure 4B).

**FIGURE 4:**
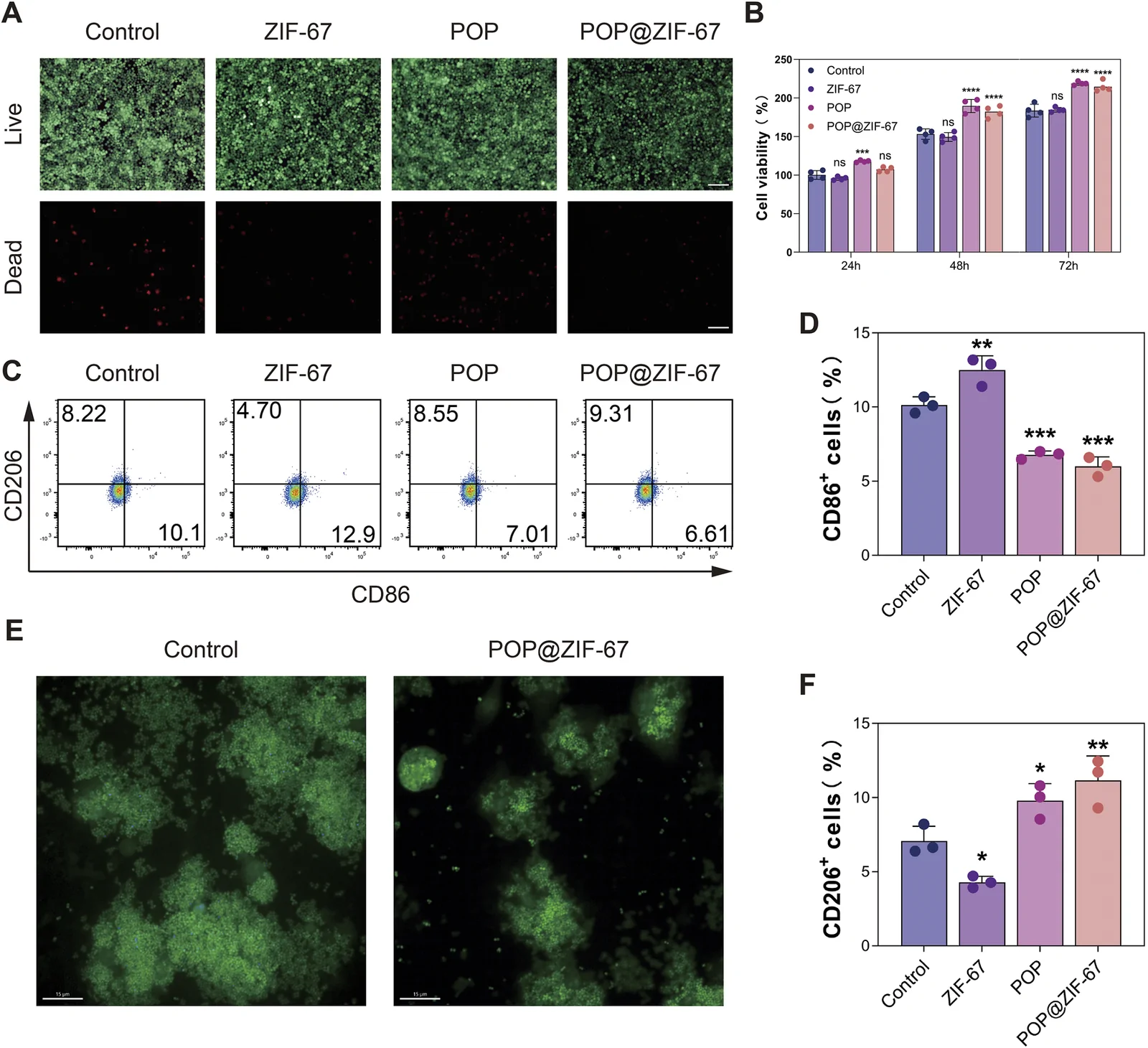
Effects of POP@ZIF-67 on the viability, polarization, and antibacterial function of RAW264.7 macrophages under infection stress. **(A)** Live/dead staining images of RAW264.7 cells. **(B)** CCK-8 viability assay of RAW264.7 cells. **(C)** Flow cytometric analysis of CD86 and CD206 expression in RAW264.7 cells. **(D)** Quantitative analysis of the proportion of CD86^+^ cells. **(E)** Representative images of Macrophage–bacterium association assay. **(F)** Quantitative analysis of the proportion of CD206^+^ cells. Data are expressed as mean ± SD, n = 3. ns, not significant; *p < 0.05, **p < 0.01, ***p < 0.001, ****p < 0.0001.

Flow cytometry was used to detect the expression of CD86 and CD206 in RAW264.7 cells. The results showed that the Control group, following stimulation with inactivated *S. aureus*, already exhibited a certain degree of the CD86^+^ pro-inflammatory phenotype. Compared with the Control group, the proportion of CD86^+^ cells in the ZIF-67 group was further elevated, suggesting that treatment with ZIF-67 alone enhances M1-like pro-inflammatory polarization of RAW264.7 cells under infection stress (Figures 4C,D). This finding suggests that although ZIF-67 possesses good antibacterial and carrier properties, its use alone may carry the risk of exacerbating the local inflammatory response. Therefore, regulating the immunostimulatory activity of ZIF-67 is key to enhancing its suitability for the treatment of bone infections. In contrast to the ZIF-67 group, the proportion of CD86^+^ cells in the POP group was significantly reduced, indicating that POP can effectively alleviate infection-stress-induced pro-inflammatory polarization. The proportion of CD86^+^ cells in the POP@ZIF-67 group was significantly lower than that in the ZIF-67 group and also lower than that in the infection-stress control group (Figures 4C,D). This indicates that when POP is loaded onto ZIF-67, it can significantly neutralize or buffer the pro-inflammatory tendency of ZIF-67, causing the composite material to no longer act as a purely pro-inflammatory stimulus but rather to exhibit milder immunomodulatory characteristics. Changes in the proportion of CD206^+^ cells further support this conclusion. The proportion of CD206^+^ cells in the ZIF-67 group was reduced, indicating that treatment with ZIF-67 alone does not promote the transition of RAW264.7 cells to a repair-associated M2-like phenotype. The proportion of CD206^+^ cells was significantly elevated in the POP and POP@ZIF-67 groups (Figures 4C,F). The antibacterial clearance capacity of macrophages was further assessed through interaction experiments between RAW264.7 cells and *S. aureus*. The results showed that while the Control group exhibited a high level of bacterial aggregation, treatment with POP@ZIF-67 enhanced contact, adhesion, and clearance between macrophages and bacteria, with a reduction in residual bacterial clusters. This suggests that POP@ZIF-67 can enhance the adhesion and killing capacity of macrophages against *S. aureus* (Figure 4E). (Alemie et al., 2025; Long et al., 2025)

To further distinguish between initial bacterial uptake by macrophages and subsequent intracellular killing, we separately measured the initial internalized bacterial load (CFU_0_) after extracellular bacterial clearance and the viable intracellular bacterial load (CFU_12h_) after an additional 12 h of culture. The bacterial phagocytosis assay showed that there were some differences in the initial internalized bacterial load in RAW264.7 cells treated with different materials (Supplementary Figure S7). Further quantification of viable intracellular bacteria revealed that the POP@ZIF-67 group had the lowest number of viable intracellular bacteria at 12 h; the intracellular CFU count in the ZIF-67 group was lower than that in the POP group (Supplementary Figure S8). Considering that the endpoint CFU count is influenced by both the initial internalized bacterial load and subsequent intracellular killing, we further calculated the relative intracellular bacterial killing efficiency based on the CFU_0_ of each group. The normalized results showed no significant difference in relative intracellular killing efficiency between the ZIF-67 group and the Control group; the relative killing efficiency of the POP group was numerically higher than that of the ZIF-67 group, while the relative killing efficiency of the POP@ZIF-67 group was significantly higher than that of both the Control and ZIF-67 groups (Supplementary Figure S9). Notably, although the number of viable intracellular bacteria in the POP group at 12 h was higher than that in the ZIF-67 group, its normalized intracellular killing efficiency was numerically higher. These results suggest that the reduction in bacterial load induced by ZIF-67 may rely more on the material’s direct antibacterial activity, whereas the effect of POP is more likely related to the enhancement of macrophage-mediated intracellular bacterial clearance. Thus, POP@ZIF-67 may combine the direct antibacterial activity of ZIF-67 with POP-mediated regulation of macrophage function, thereby producing a complementary bacterial clearance effect.

In summary, ZIF-67 treatment alone exhibited a strong pro-inflammatory tendency under infection stress conditions, specifically manifested as an increased proportion of CD86^+^ cells and a decreased proportion of CD206^+^ cells; whereas the introduction of POP significantly mitigated this pro-inflammatory effect. POP@ZIF-67 reduces CD86^+^ M1-like polarization and enhances CD206^+^ repair-related polarization while maintaining good cellular compatibility and the antimicrobial clearance capacity of macrophages. This demonstrates that the POP loading strategy can further improve the material’s immunomodulatory properties while preserving the antimicrobial and framework functions of ZIF-67.

### 2.5 POP@ZIF-67 regulates BMDM polarization and inhibits the secretion of infection-stress-related inflammatory factors

Primary bone marrow-derived macrophages (BMDMs) co-cultured with inactivated *S. aureus* were used to further validate the regulation of phenotypic polarization. Macrophages can switch to either a pro-inflammatory M1-like or a reparative M2-like phenotype under different microenvironmental stimuli, and factors such as the size, surface charge, composition, and release behavior of nanomaterials can all influence macrophage polarization. Therefore, material-mediated macrophage reprogramming has become an important strategy in infection control and tissue repair (Dahri et al., 2025; Luo et al., 2024; Wang et al., 2021). Immunofluorescence (IF) staining was used to examine the expression of CD86 and CD206 in BMDMs following different treatments. The CD86 fluorescence intensity in the ZIF-67 group was further enhanced compared to the infection-stress group, indicating that treatment with ZIF-67 alone exacerbates the pro-inflammatory phenotype of BMDMs under infection stress. The CD86 signal was significantly reduced in the POP group, while the CD86 fluorescence intensity in the POP@ZIF-67 group was significantly lower than that in the ZIF-67 group, indicating that POP loading effectively mitigates the pro-inflammatory response induced by ZIF-67 (Figures 5A,B). Regarding changes in CD206 expression, CD206 expression was significantly increased in the POP-containing group, suggesting that when POP is loaded onto ZIF-67, the composite material retains the functional properties of the ZIF-67 framework while maintaining strong M2-like immunomodulatory capacity (Figure 5C). (Chen et al., 2025) Quantitative analysis showed that *Il6* expression was approximately 9.5, 12.5, 0.8, and 3.0 relative units in the Control, ZIF-67, POP, and POP@ZIF-67 groups, respectively, whereas *Il10* expression was approximately 7.5, 1.0, 20.0, and 15.5 relative units, respectively (Figures 5D,E). The concentrations of IL-1β were approximately 1.4, 1.7, 0.25, and 0.5 relative units, and those of TNF-α were approximately 1.55, 1.65, 0.30, and 0.55 relative units, respectively (Figures 5F,G). Compared with ZIF-67, POP@ZIF-67 significantly reduced the levels of pro-inflammatory factors (*P* < 0.001) while maintaining increased Il10 expression (Wang et al., 2025).

**FIGURE 5:**
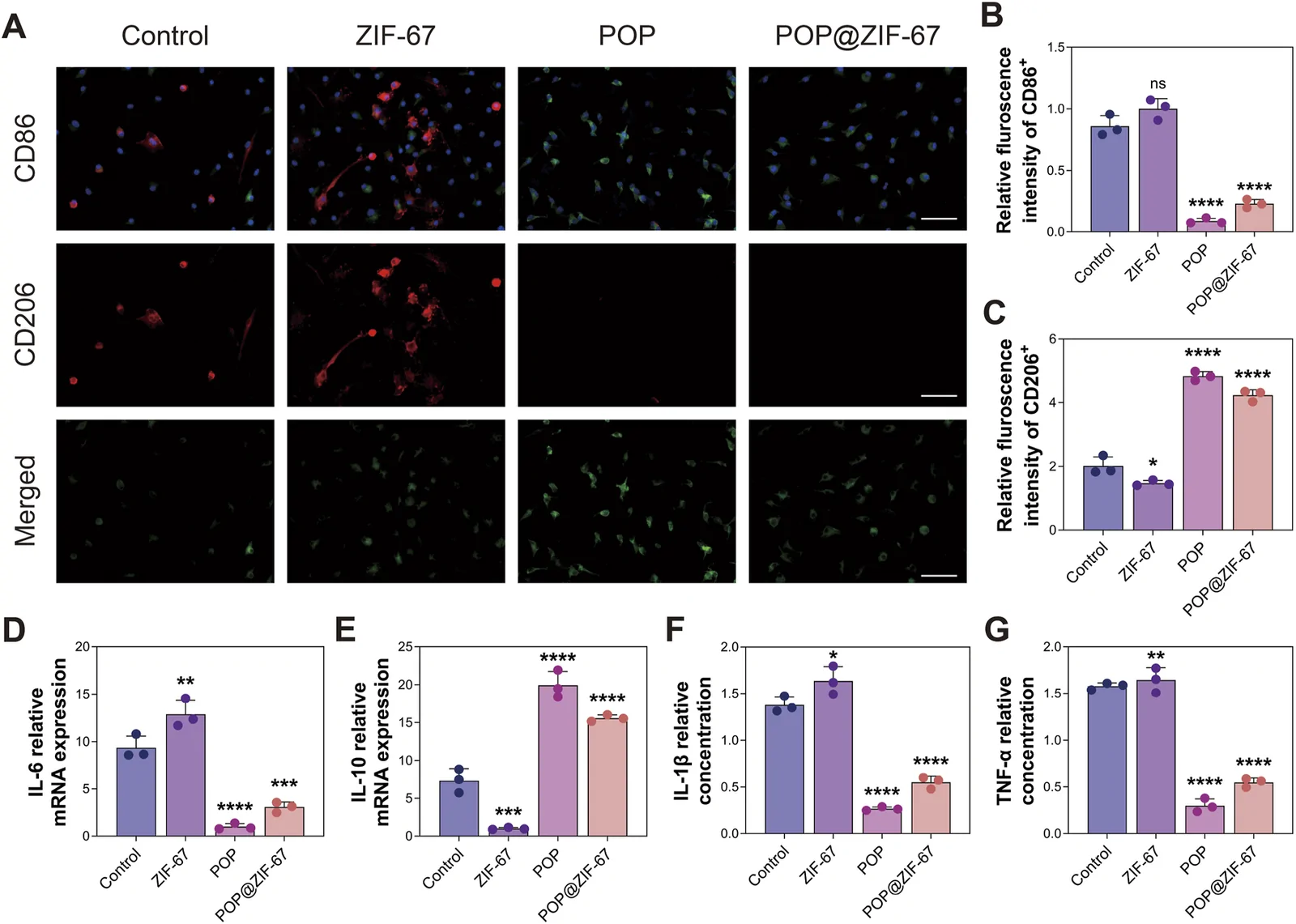
Effects of POP@ZIF-67 on the polarization and inflammatory cytokine expression of BMDMs under infection stress. **(A)** IF staining images of CD86/CD206 in BMDMs. **(B)** Quantitative analysis of CD86 fluorescence intensity. **(C)** Quantitative analysis of CD206 fluorescence intensity. **(D)** Relative expression levels of *Il6* mRNA. **(E)** Relative expression levels of *Il10* mRNA. **(F)** ELISA for IL-1β in BMDM culture supernatants. **(G)** ELISA for TNF-α in BMDM culture supernatants. Data are presented as mean ± SD, n = 3. ns, not significant; *p < 0.05, **p < 0.01, ***p < 0.001, ****p < 0.0001.

In summary, the BMDMs experiment further validated the immunomodulatory capacity of POP@ZIF-67. While retaining the functional properties of the ZIF-67 framework, POP@ZIF-67 mitigates the pro-inflammatory effects of ZIF-67 through POP, thereby reducing the release of pro-inflammatory cytokines and promoting the expression of CD206 and *Il10*.

### 2.6 POP@ZIF-67 reduces the bacterial load in bone marrow *in vivo* and improves the immune imbalance associated with chronic osteomyelitis

The *in vivo* therapeutic effects of POP@ZIF-67 were further evaluated in a mouse model of chronic osteomyelitis (Liu et al., 2025; Su et al., 2025). In this experiment, the COM group served as the chronic osteomyelitis model group, while the ZIF-67 and POP@ZIF-67 groups were intervention groups that received the corresponding treatments based on the COM model. Changes in splenic immune status after treatment were evaluated using flow cytometry. Following ZIF-67 treatment, the proportion of CD86^+^ cells within the CD11b^+^F4/80^+^ splenic macrophage population decreased, which may be partly related to the reduction in bacterial burden at the infection site. In the POP@ZIF-67 group, the proportion of CD86^+^ macrophages decreased further, while the proportion of CD206^+^ macrophages increased significantly, suggesting a shift toward a less pro-inflammatory and more repair-associated macrophage phenotype in the spleen (Figures 6A,D,E). The residual bacterial levels in the bone marrow cavities of mice in each group were assessed using spread plate assays of bone marrow samples. Numerous bacterial colonies were observed on the plates from the COM group, whereas the number of colonies on the plates from the ZIF-67 and POP@ZIF-67 groups was significantly reduced (Figure 6B). Quantitative analysis showed that both ZIF-67 and POP@ZIF-67 significantly reduced bacterial loads in bone marrow tissue (Figure 6C). This indicates that POP@ZIF-67 can effectively reduce the bacterial burden associated with chronic osteomyelitis *in vivo* (Yang et al., 2025; Zhou et al., 2025). Previous studies have shown that infection-related bone repair requires first reducing the pathogen burden, followed by promoting the transition of macrophages from a pro-inflammatory state to a reparative state, in order to prevent chronic inflammation from continuously inhibiting bone regeneration (Liu et al., 2025; Chen et al., 2026).

**FIGURE 6:**
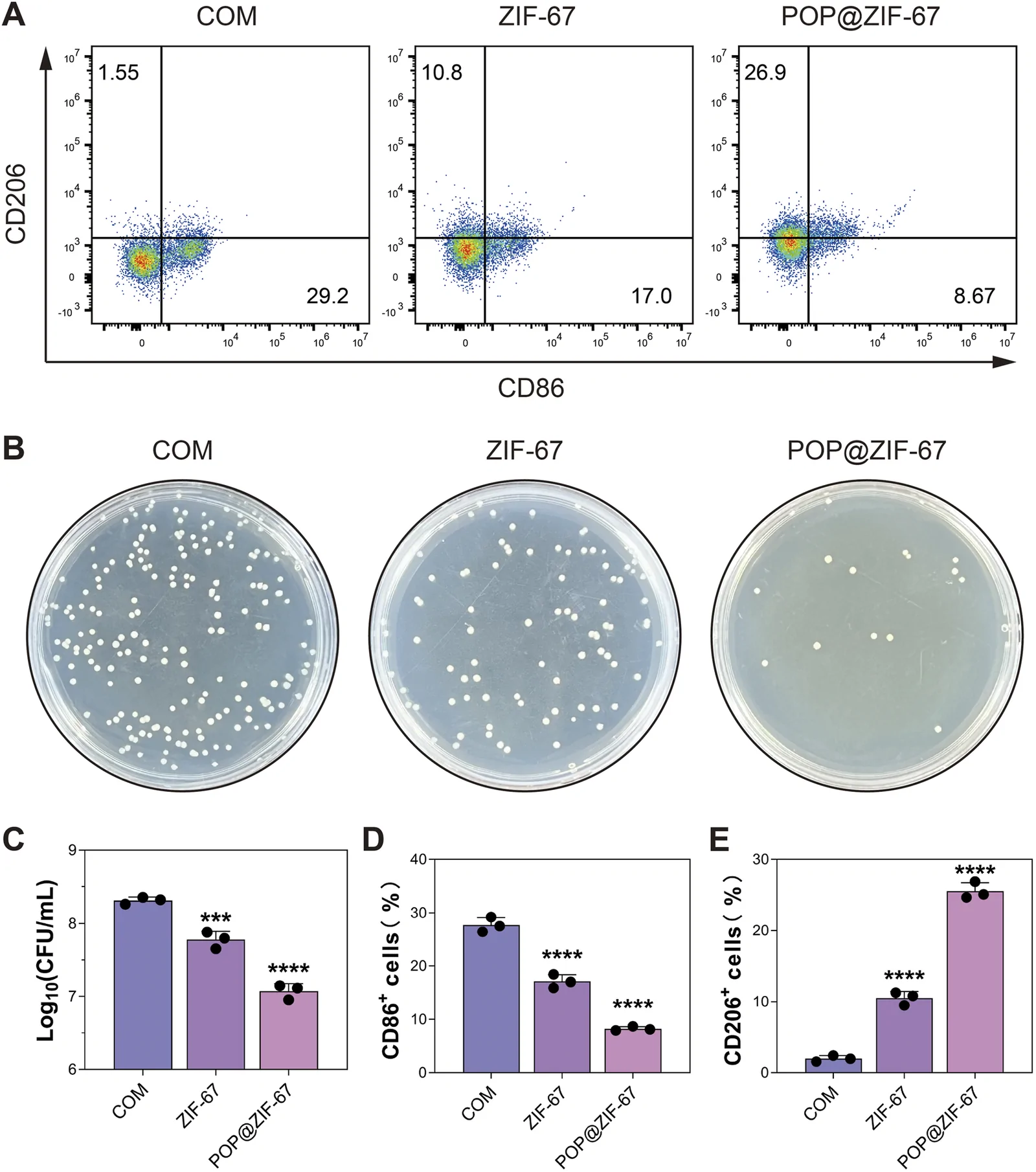
Effects of POP@ZIF-67 on bacterial load and splenic macrophage phenotype in mice with chronic osteomyelitis. **(A)** Flow cytometric analysis of CD86/CD206 expression in splenic cells. **(B)** Representative image of bacterial plate counts in mouse bone marrow after treatment. **(C)** Quantitative analysis of bacterial colonies in bone marrow tissue. **(D)** Quantitative analysis of the proportion of CD86^+^ cells in the spleen. **(E)** Quantitative analysis of the proportion of CD206^+^ cells in the spleen. Data are presented as mean ± SD, n = 3. ***p < 0.001, ****p < 0.0001.

To further evaluate the regulatory effect of POP@ZIF-67 on the local immune microenvironment of infected bone, we performed iNOS/TGF-β IF staining on bone marrow tissue from the infected tibia following treatment. The results showed that, compared with the COM group, POP@ZIF-67 treatment significantly improved the inflammatory state within the bone marrow of the infected region, reduced the expression of pro-inflammatory markers, and enhanced the expression of immune markers associated with tissue repair (Supplementary Figure S10). These findings further demonstrate that POP@ZIF-67 not only modulates the state of peripheral immune cells but also improves the local immune microenvironment of infected bone, providing evidence at the local tissue level to support its role in promoting infection control and bone structural repair.

These results demonstrate that POP@ZIF-67 not only exerts antibacterial effects *in vivo* but also modulates the dysregulated immune microenvironment in chronic osteomyelitis, providing a more favorable microenvironmental foundation for subsequent bone tissue structural repair and osteogenic regeneration.

### 2.7 POP@ZIF-67 improved local inflammation, residual bacteria, and tissue repair in the bone marrow of mice with chronic osteomyelitis

Bone marrow tissue was analyzed using H&E, Giemsa, and Masson staining. H&E staining revealed extensive, dense inflammatory cell infiltration in the bone marrow tissue of the COM group, with disruption of the local tissue structure. In the ZIF-67 group, inflammatory cell infiltration was slightly reduced compared to the COM group, while in the POP@ZIF-67 group, inflammatory cell infiltration was further reduced and the bone marrow tissue structure was clearer, suggesting that POP@ZIF-67 can more effectively alleviate the local inflammatory state associated with chronic osteomyelitis (Figure 7A). (Lu et al., 2024; Tian et al., 2024) Giemsa staining results showed that the COM group exhibited a relatively high number of Giemsa-positive bacterial signals, while the ZIF-67 group showed a significant reduction in positive bacterial signals; no obvious Giemsa-positive bacterial signals were observed in the POP@ZIF-67 group, suggesting that the composite material can more thoroughly reduce residual bacterial within the medullary cavity (Figure 7B). (Lu et al., 2024; Yang et al., 2024) Masson staining was used to evaluate local collagen deposition and tissue repair. The COM group showed minimal deposition of blue-stained collagen fibers, suggesting that persistent infection and inflammation inhibited local matrix remodeling and tissue repair. Compared to the COM group, the ZIF-67 group showed more collagen fibers, while the POP@ZIF-67 group exhibited the most with a more continuous distribution, suggesting that it possesses the strongest ability to promote collagen formation and tissue repair (Figure 7C). This phenomenon can be explained in two ways: first, POP@ZIF-67 is more effective at reducing residual bacteria, thereby mitigating the inhibitory effects of infection on local bone marrow repair; second, the POP loading buffers the pro-inflammatory tendencies of ZIF-67, improving the immune microenvironment required for tissue repair.

**FIGURE 7:**
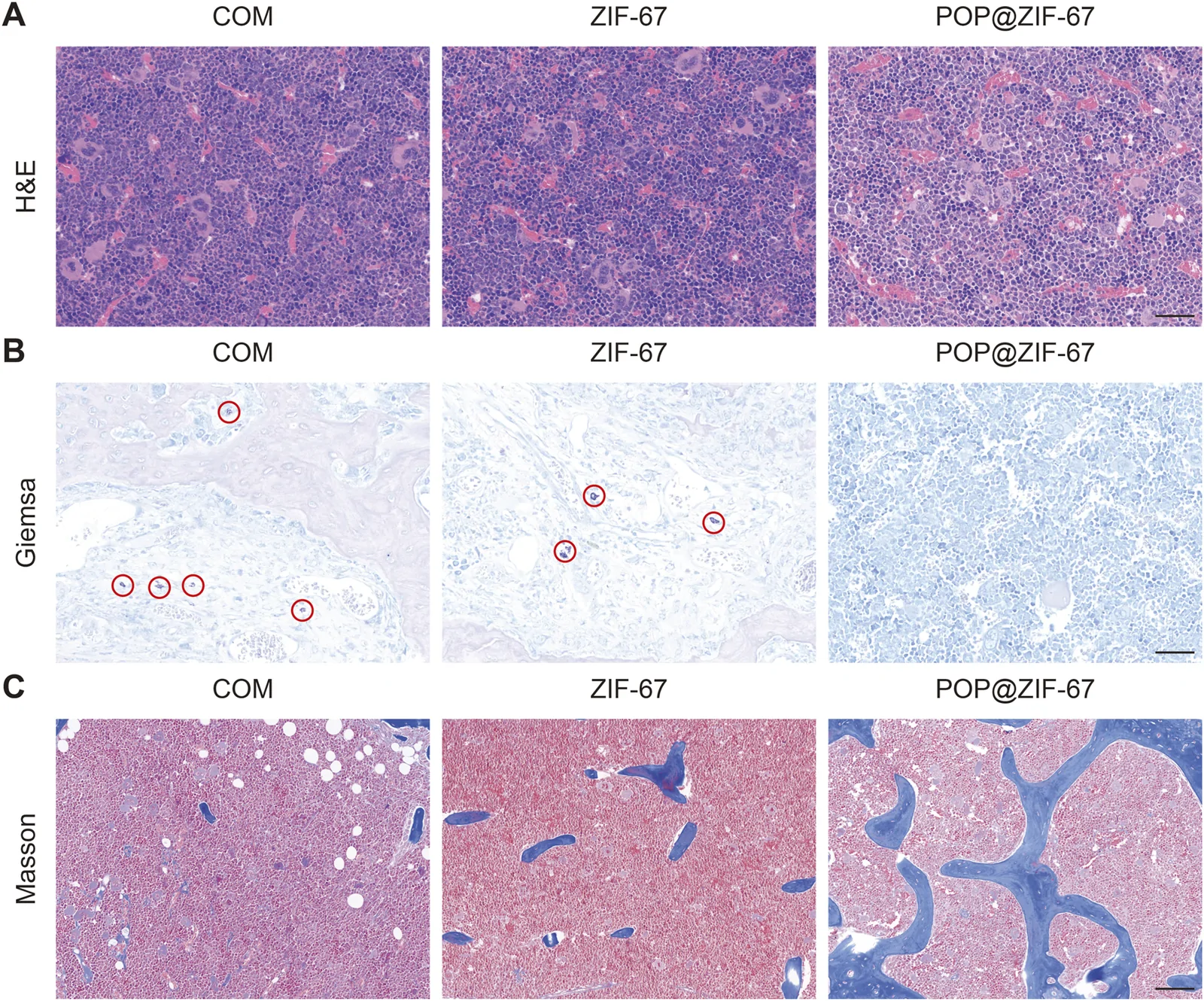
Effects of POP@ZIF-67 on pathological changes in bone marrow tissue of mice with chronic osteomyelitis. **(A)** H&E-stained image of mouse bone marrow tissue. Scale bar, 50 μm. **(B)** Giemsa-stained image of mouse bone marrow tissue. Scale bar, 50 μm. **(C)** Masson-stained image of mouse bone marrow tissue. Scale bar, 100 μm.

### 2.8 POP@ZIF-67 promotes bone structural repair in the tibiae of mice with chronic osteomyelitis

Micro-CT was used to verify the ability of POP@ZIF-67 to promote bone repair at the bone structural level (Qin et al., 2024; Shen et al., 2024). The COM group exhibited significant localized bone destruction in the tibia, with poor cortical continuity and sparse trabecular structure, indicating that chronic osteomyelitis caused severe bone loss and structural disruption. Compared to the COM group, the ZIF-67 group showed slightly better bone structural recovery, with improvements in cortical contour and trabecular structure. In the POP@ZIF-67 group, the tibial cortical bone was more continuous and the trabecular structure was denser, suggesting a stronger ability to promote bone repair (Figure 8A). To further enhance the comparability and visualization of micro-CT results, standardized 3D reconstructions of the tibial structure were performed using anatomically consistent orientation and scanning parameters across all groups. The reconstructed images consistently revealed differences in tibial structural integrity among the treatment groups, with the POP@ZIF-67 group showing better preservation of bone morphology compared to the COM and ZIF-67 groups (Supplementary Figure S11). Quantitative micro-CT analysis showed that BMD values were approximately 0.12, 0.22, and 0.31 g HA/cm^3^ in the COM, ZIF-67, and POP@ZIF-67 groups, respectively (Figure 8B). The corresponding BV/TV values were approximately 22%, 40%, and 57%, while Tb.N values were approximately 0.55, 1.03, and 1.37 mm^-1^, respectively (Figures 8C,D). Tb.Th values were approximately 0.24, 0.30, and 0.37 mm in the COM, ZIF-67, and POP@ZIF-67 groups, respectively (Figure 8E). Compared with the COM group, POP@ZIF-67 significantly improved all measured bone microstructural parameters (*P* < 0.0001), indicating enhanced structural repair of the infected tibia (Sun et al., 2024; Sun et al., 2025).

**FIGURE 8:**
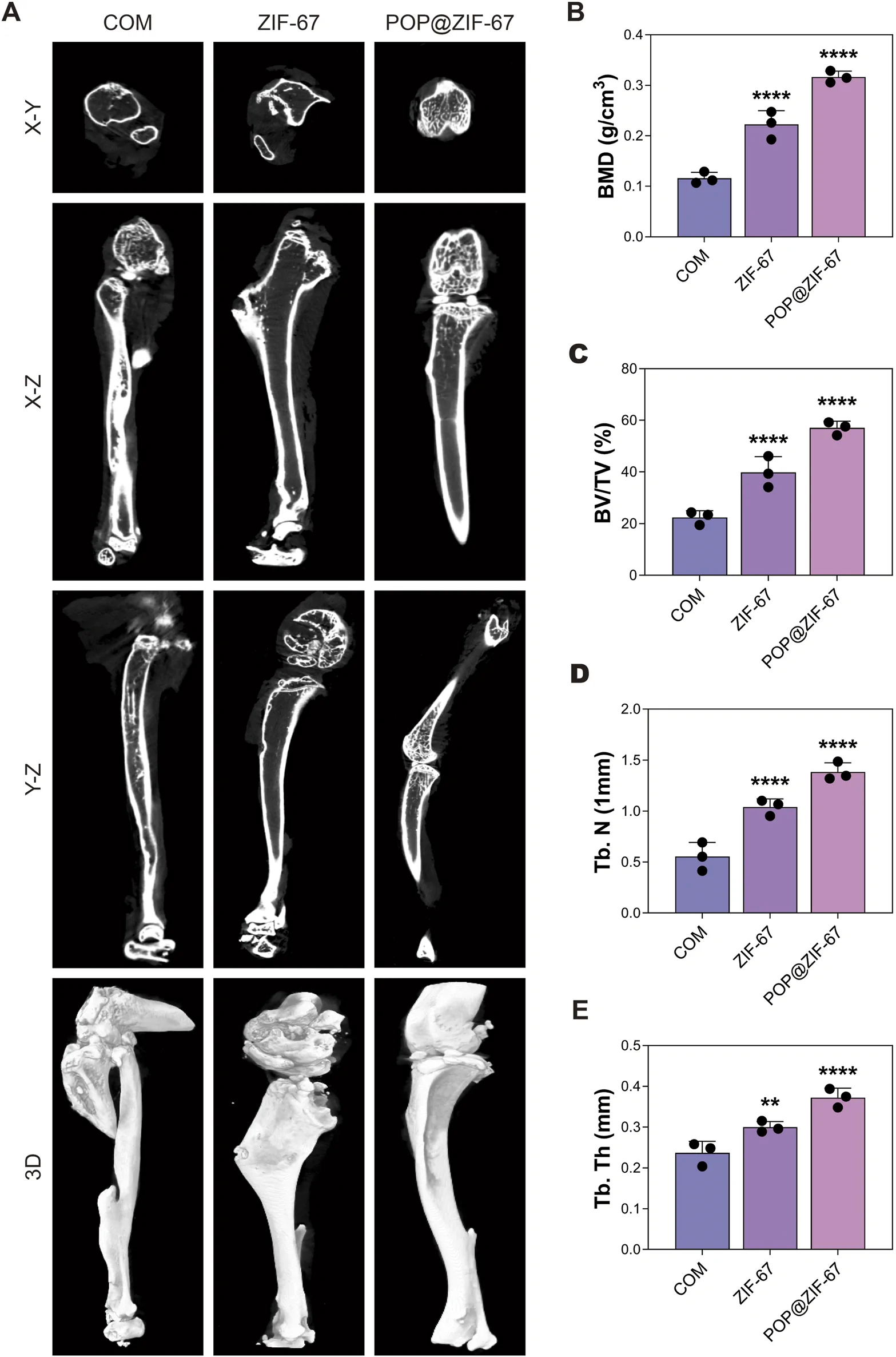
Effect of POP@ZIF-67 on tibial bone remodeling in mice with chronic osteomyelitis. **(A)** Representative micro-CT images of the mouse tibia, including cross-sections in the X-Y, X-Z, Y-Z directions and representative 3D reconstruction images. **(B)** Quantitative analysis of BMD. **(C)** Quantitative analysis of BV/TV. **(D)** Quantitative analysis of Tb.N. **(E)** Quantitative analysis of Tb.Th. Data are presented as mean ± SD, n = 3. **p < 0.01, ****p < 0.0001.

Based on the combined micro-CT images and bone morphometric results, ZIF-67 monotherapy demonstrated a certain degree of osteogenic effect, which may be related to its *in vivo* antibacterial action that reduces the inflammatory stimulus caused by infection; however, the bone repair effect of POP@ZIF-67 was significantly superior to that of ZIF-67 monotherapy, indicating that the introduction of POP further improved the bone repair microenvironment (Qin et al., 2024; Sun et al., 2025).

### 2.9 POP@ZIF-67 promotes OCN expression *in vivo* and exhibits good biocompatibility

OCN is an important non-collagenous protein secreted by osteoblasts and is generally regarded as a key marker of bone formation, bone matrix maturation, and mineralization (Martiniakova et al., 2024; Zhu et al., 2024). Immunohistochemical results for OCN showed that OCN-positive expression was weak in the bone marrow tissue of the COM group, suggesting that bone formation and bone matrix maturation are suppressed in chronic osteomyelitis. The OCN-positive signal in the ZIF-67 group was stronger than that in the COM group, indicating that ZIF-67 can improve the bone repair microenvironment to some extent after reducing the local infection burden. The POP@ZIF-67 group exhibited the most pronounced OCN-positive staining; combined with quantitative analysis, this suggests that it possesses a stronger capacity to promote osteogenesis and bone matrix maturation (Figure 9A; Supplementary Figure S12). (Li et al., 2026; Li et al., 2025) To evaluate the *in vivo* biosafety of POP@ZIF-67, H&E staining analysis was performed on major organs. The results showed that after treatment with POP@ZIF-67, no significant tissue structural damage, necrosis, or abnormal infiltration of inflammatory cells was observed in the major organs of the mice, including the heart, liver, spleen, lungs, and kidneys (Figure 9B). These findings indicate that, under the current treatment conditions, POP@ZIF-67 did not cause significant pathological damage to major organs and exhibits good *in vivo* tissue compatibility.

**FIGURE 9:**
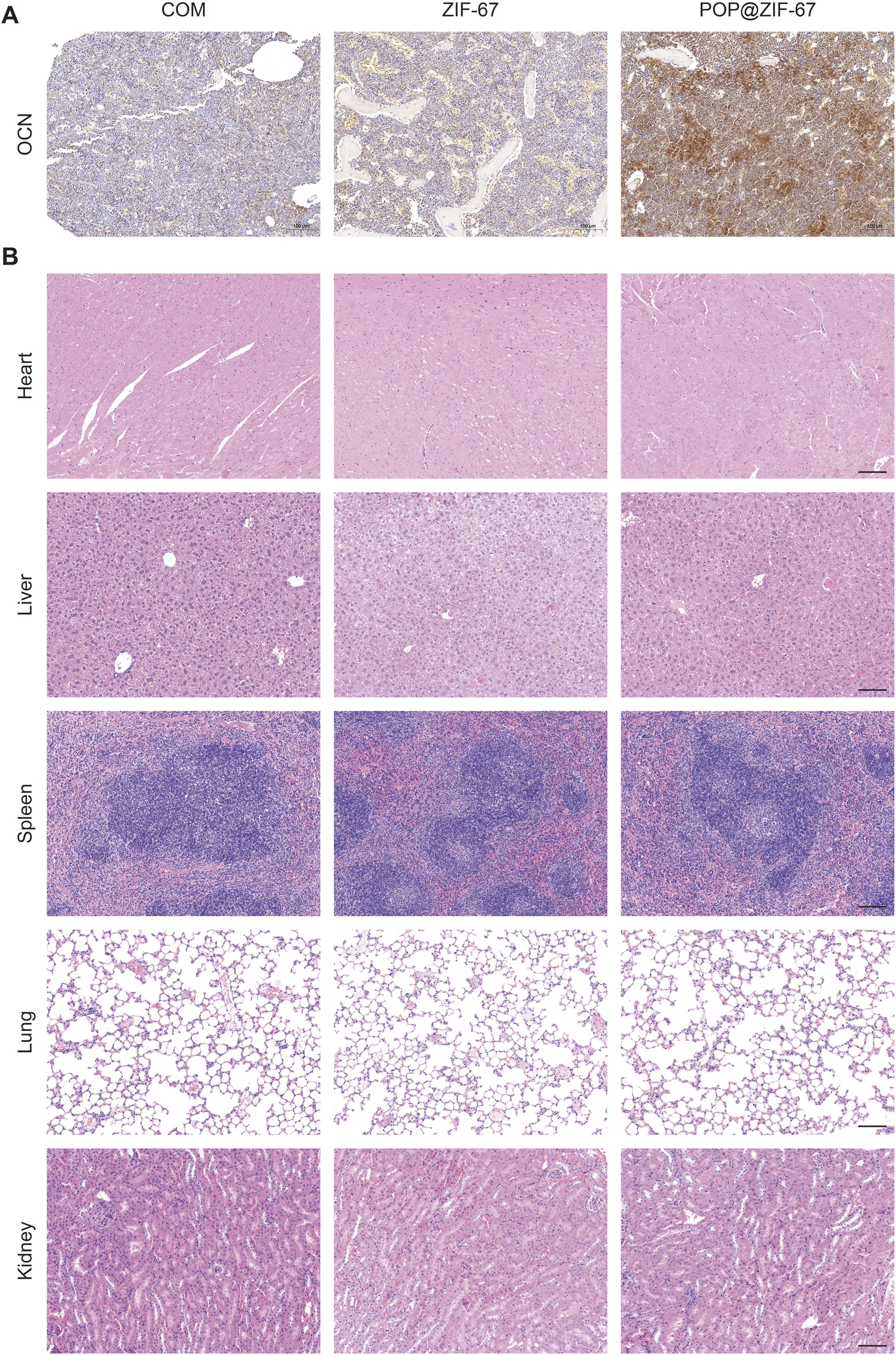
Effects of POP@ZIF-67 on OCN expression in the bone marrow of mice with chronic osteomyelitis and on *in vivo* biosafety. **(A)** Immunohistochemical staining images of OCN in mouse bone marrow tissue. **(B)** H&E-stained images of major organs (heart, liver, spleen, lungs, and kidneys) in mice after POP@ZIF-67 treatment. Scale bar, 100 μm.

## 3 Discussion

The challenge in treating chronic osteomyelitis lies not merely in “killing bacteria,” but in the pathological cycle of mutual restraint that forms among infection, inflammation, and bone regeneration. Persistently colonizing bacteria and biofilms keep local inflammation at high levels, while the long-term pro-inflammatory microenvironment hinders the repair of infection-induced bone defects. Therefore, in recent years, the design of materials for treating bone infections has gradually shifted from a single antimicrobial strategy to an integrated therapeutic approach combining “antimicrobial action, immune regulation, and bone regeneration.” Research on materials that combine immunomodulatory and bone repair functions also suggests that materials must not only influence bacteria and osteoblasts but also actively regulate the behavior of immune cells in order to establish a more suitable microenvironment for bone regeneration (Li et al., 2025; Zhang et al., 2025).

In this study, ZIF-67 served as the primary antimicrobial framework. ZIF-67 possesses cobalt-based metal nodes, a porous structure, and acid-responsive properties, and may exert antibacterial effects through metal ion-related mechanisms, surface contact, and oxidative stress (Zhang et al., 2025; Martín-Martínez et al., 2025). However, it should be noted that antimicrobial activity alone is not sufficient for the treatment of chronic osteomyelitis. *In vitro* results suggest that ZIF-67 treatment alone still exhibits a certain pro-inflammatory tendency in macrophages, indicating that its antimicrobial activity may be accompanied by immunostimulatory effects. Therefore, the key challenge with ZIF-67 is how to further control the inflammatory amplification effects it may trigger in the infected microenvironment while retaining its original antimicrobial function.

The introduction of POP is specifically intended to address this challenge. The role of POP in the composite system is more akin to that of an “immune buffer.” Experiments with RAW 264.7 and BMDMs collectively demonstrated that POP reduced pro-inflammatory markers and increased repair-related markers such as CD206 and IL-10. Natural polysaccharides are believed to influence macrophage responses by regulating inflammatory pathways such as TLR4/NF-κB, and acidic heteropolysaccharides derived from purslane have also been reported to inhibit macrophage-related inflammatory damage in inflammatory models (Yang et al., 2025; Li et al., 2025). Therefore, the advantage of POP@ZIF-67 is not simply to enhance the existing antibacterial function of ZIF-67, but rather to utilize the anti-inflammatory regulatory function of POP, transforming the composite material into a platform that integrates antibacterial activity with immunomodulatory properties.

This “antimicrobial framework + immune buffering” design concept has also been validated by *in vivo* experimental results. By the 4-week endpoint, the ZIF-67 group had already shown a certain degree of inflammation resolution and a trend toward bone repair, which may primarily stem from reduced persistent infection stimulation following the reduction in bacterial load. In contrast, POP@ZIF-67 not only further reduced residual bacteria in the bone marrow but also demonstrated superior repair effects in histological experiments. Recent studies on antimicrobial-immune adaptive bone repair frameworks have also indicated that bacterial control and immune regulation jointly provide a more favorable regenerative microenvironment; simultaneously, research in bone immunology emphasizes that the interaction between macrophages and biomaterials directly influences the subsequent quality of bone healing (Qian et al., 2024; Wang et al., 2026). Therefore, the therapeutic advantages of POP@ZIF-67 should be understood as a holistic remodeling of the pathological microenvironment of chronic osteomyelitis, rather than an improvement in a single therapeutic criterion.

Mechanistically, the antibacterial activity of ZIF-67 may involve cobalt-ion release, disruption of bacterial membranes, and ROS-related oxidative damage; nanoenzyme-based systems have also been reported to generate ROS that damage bacterial membranes, proteins, and DNA (Zhou et al., 2024; Li et al., 2025). Because the extracellular polymeric matrix of biofilms limits antimicrobial penetration and increases bacterial resistance, antibiofilm activity is particularly relevant to bone infection treatment (Xie et al., 2025; Gu et al., 2024). This integrated strategy is consistent with previous ZIF-based anti-infective interfaces that combine antibacterial activity with immunoregulation to improve osseointegration (Dong et al., 2024; Ding et al., 2023). Consistent with this concept, the present histological findings showed reduced bacterial residue and inflammatory infiltration, together with increased collagen deposition, after POP@ZIF-67 treatment, which may contribute to its improved tissue-repair effect (Tian et al., 2024; Gupta et al., 2024; Zhou et al., 2024).

Overall, this study proposes a strategy for constructing the POP@ZIF-67 composite material for the treatment of chronic osteomyelitis. POP incorporation was associated with reduced pro-inflammatory macrophage responses alongside antibacterial activity and improved bone repair. These findings support further investigation of this combined treatment strategy for infectious bone defects.

Several limitations should be considered. The *in vivo* study did not include POP-only, healthy or sham-operated, or standard antibiotic control groups, and dynamic bone formation was not assessed; these additions would further clarify the component-specific and regenerative effects of the treatment. In addition, splenic flow-cytometric analysis and non-macrophage-specific iNOS/TGF-β staining support systemic immune and local microenvironmental changes but do not establish macrophage reprogramming within infected bone. The observed immunomodulatory effects are therefore interpreted at the phenotypic level, while the contributions of TLR4/NF-κB, ERK/NF-κB, Co^2+^ release, ROS generation, POP dose, and release kinetics remain to be clarified. Finally, the 4-week observation period and repeated intramedullary injection protocol do not fully address long-term degradation, cobalt biodistribution, delayed toxicity, or clinical administration. Further studies will help define the mechanism, safety, and translational potential of POP@ZIF-67.

## 4 Conclusion

POP@ZIF-67 was successfully constructed as a multifunctional platform for chronic osteomyelitis treatment. ZIF-67 provided effective antibacterial and antibiofilm activity, while POP partially neutralized the pro-inflammatory tendency of ZIF-67 and promoted macrophage polarization toward a repair-associated phenotype. This combined effect reduced bacterial burden, alleviated local inflammation, enhanced collagen deposition, and promoted osteogenic repair *in vivo*. Above all, POP@ZIF-67 achieved coordinated antibacterial, immunomodulatory, and pro-osteogenic effects, offering a promising strategy for restoring the infected bone microenvironment and improving chronic osteomyelitis repair.

## 5 Materials and methods

### 5.1 Materials and reagents

2-Methylimidazole (C_4_H_6_N_2_, 98%) and cobalt nitrate hexahydrate (Co(NO_3_)_2_·6H_2_O, 99%) were purchased from Aladdin Chemistry Co., Ltd. Methanol (MeOH, 99.7%) was obtained from Sinopharm Chemical Reagent Co., Ltd. Portulaca oleracea polysaccharide (POP) was purchased from Solarbio Life Sciences (Beijing, China). According to the quality inspection report provided by the manufacturer, this product is a white to off-white solid with a purity of 90.26% and a loss on drying of 1.20%; it is soluble in water to form a colorless, transparent solution (2 mg/mL). See the attachment for relevant quality information.

### 5.2 Synthesis of ZIF-67

Under magnetic stirring, 1.314 g of 2-methylimidazole was dissolved in 32 mL of methanol to yield a clear ligand solution. Separately, dissolve 1.164 g of cobalt (NO_3_)_2_·6H_2_O in another 32 mL of methanol. Then, slowly add the cobalt nitrate solution to the 2-methylimidazole solution and stir the mixture continuously at room temperature for 15 min. The reaction mixture was then allowed to stand overnight to permit crystal growth. The purple precipitate was collected by centrifugation, washed three times with methanol to remove residual reactants, and finally vacuum-dried at 80 °C for 12 h to yield ZIF-67 powder (Yong et al., 2023).

### 5.3 Synthesis of POP@ZIF-67

Dissolve 10 mg of POP powder in deionized water. Then, weigh out 20 mg of ZIF-67 and dissolve it in the POP solution. Stir magnetically for 1 day. After collection, wash the material three times with deionized water and vacuum-dry it to obtain the POP@ZIF-67 solid powder.

### 5.4 Characterization of POP@ZIF-67

The morphology and nanoscale structure of the materials were examined using a transmission electron microscope (TEM, Hitachi H7650). Fourier-transform infrared (FTIR) spectroscopy of ZIF-67, POP, and POP@ZIF-67 was performed using a Nicolet IS50 spectrometer (Thermo Fisher Scientific). Hydrodynamic diameter, particle size distribution, and ζ-potential were determined using a NanoZS ZEN3600 instrument (Malvern Instruments, UK). Nitrogen adsorption-desorption measurements were performed on an ASAP 2460 analyzer to evaluate the materials’ porous structures. Specific surface area was calculated from the adsorption isotherms using the Brunauer-Emmett-Teller (BET) method, and pore size distribution was determined using the Barrett–Joyner–Halenda (BJH) model.

### 5.5 *In vitro* antimicrobial assay

The antimicrobial activity of different samples against *S. aureus* ATCC 25923 was evaluated by CFU counting and live/dead staining. In brief, bacteria in the logarithmic phase were adjusted to a total colony count of 1.0 × 10^7^ per sample and incubated with the corresponding samples. After treatment, the bacterial suspensions were serially diluted and spread onto LB agar plates, which were then incubated overnight at 37 °C for CFU counting. For live/dead staining, the treated bacteria were stained with SYTO 9/PI and observed using a confocal laser scanning microscope. To evaluate the effect of antimicrobial agents on biofilms, *S. aureus* biofilms were established in confocal culture dishes and then treated with different samples. The biofilms were stained with SYTO 9/PI and observed using CLSM.

### 5.6 Cell culture

MC3T3-E1 cells, RAW264.7 macrophages, and bone marrow-derived macrophages (BMDMs) were used in this study. MC3T3-E1 (Procell, Wuhan, China) cells and RAW264.7 (Procell, Wuhan, China) cells were cultured in complete medium containing 10% fetal bovine serum (FBS, Procell, Wuhan, China) and 1% penicillin-streptomycin. Cells were maintained at 37 °C in a humidified incubator with 5% CO_2_. BMDMs were isolated from the femurs and tibias of *C57BL/6* mice. Bone marrow cells were flushed out under sterile conditions and cultured in α-MEM supplemented with 10% fetal bovine serum, 1% penicillin-streptomycin, and 30 ng/mL macrophage colony-stimulating factor (M-CSF, ABclonal Biotechnology Co., Ltd.). The medium was refreshed every 3 days. After 7 days of induction, mature BMDMs were collected for subsequent experiments.

### 5.7 Cell viability assay

Cell viability was measured using a Cell Counting Kit-8 (CCK-8, Biosharp, Beijing, China) assay. Briefly, MC3T3-E1 cells or RAW264.7 cells were seeded into 96-well plates and allowed to adhere overnight. The medium was then replaced with fresh medium containing ZIF-67, POP, or POP@ZIF-67. After 24, 48, and 72 h of treatment, CCK-8 working solution was added to each well and incubated at 37 °C. The absorbance at 450 nm was measured using a microplate reader. Cell viability was calculated relative to the Control group.

### 5.8 Live/dead staining

RAW264.7 cells were seeded into 24-well plates and treated with different formulations. After treatment, cells were washed with PBS and stained using a Calcein-AM/PI live/dead staining kit (Servicebio, Wuhan, China) according to the manufacturer’s instructions. After incubation in the dark, the cells were washed again and observed using an inverted fluorescence microscope (ICX41, SOPTOP, China). Live cells were stained green, while dead cells were stained red.

### 5.9 *In vitro* osteogenesis

MC3T3-E1 cells were used to evaluate the osteogenic effect of POP@ZIF-67. Cells were seeded into culture plates and cultured in osteogenic induction medium. Different treatments were added during osteogenic induction. For alkaline phosphatase (ALP) staining, cells were fixed after early osteogenic induction and stained with an ALP staining kit (Beyotime, Shanghai, China). Images were captured using a light microscope, and the ALP-positive area was quantified using ImageJ software. For Alizarin Red S (ARS) staining, cells were fixed after mineralization induction and stained with ARS solution. Mineralized nodules were observed under a microscope. For quantitative analysis, the staining was extracted and measured by absorbance. The results were normalized to the Control group.

### 5.10 *In vitro* flow cytometry

RAW264.7 cells were collected after treatment and washed with cold PBS. Cells were blocked and incubated with antibodies against CD86 (PC5.5) and CD206 (FITC). After antibody incubation, the cells were washed and resuspended in PBS. Flow cytometry was performed to detect the proportions of CD86^+^ and CD206^+^ cells. Data were analyzed using FlowJo software.

### 5.11 Macrophage–bacterium association assay

RAW264.7 cells were seeded into culture plates and treated with different formulations. *Staphylococcus aureus* was then added to the cells and incubated for a defined period. After incubation, extracellular bacteria were removed by washing with PBS. Cells were fixed and observed under a microscope (Andor BC43). The bacterial uptake ability of macrophages was evaluated by comparing intracellular bacteria and extracellular free bacteria among different groups.

### 5.12 Experiment on bacterial phagocytosis and intracellular killing by macrophages

The assays were performed following previously reported methods with appropriate modifications (Lu et al., 2023). RAW264.7 macrophages were seeded at a density of 2 × 10^5^ cells/well in a 24-well plate. After the cells adhered, the medium was replaced with antibiotic-free medium, and the cells were pretreated with the corresponding materials for 12 h according to the experimental groups. The medium was then discarded, and *Staphylococcus aureus* was added at a multiplicity of infection (MOI) of 10. The cells were incubated at 37 °C for 30 min to allow the macrophages to phagocytose the bacteria. After infection, the bacteria-containing medium was discarded, and the cells were washed three times with sterile PBS. Fresh culture medium containing 20 μg/mL lysostaphin and 50 μg/mL gentamicin was then added, and the cells were incubated for an additional 1 h to eliminate residual extracellular bacteria. For the bacterial phagocytosis assay, after extracellular bacteria were eliminated, the cells were washed three times with sterile PBS, and 0.1% (v/v) Triton X-100 was immediately added to thoroughly lyse the cells. The cell lysate was collected and serially diluted with sterile PBS; dilutions of the lysate were plated onto LB agar plates, which were incubated overnight at 37 °C, followed by colony counting. The intracellular colony-forming units (CFU) measured at this point were defined as the initial internalized bacterial load (CFU_0_) and were used to evaluate the phagocytic capacity of macrophages in different treatment groups. For the intracellular killing assay, the procedures for cell seeding, material pretreatment, bacterial infection, and extracellular bacterial clearance were identical to those in the phagocytosis assay described above. After clearing extracellular bacteria, the cells were washed three times with sterile PBS, and the medium was replaced with fresh medium containing 1% penicillin-streptomycin and the corresponding treatment materials. The cells were then cultured for an additional 12 h. At the end of the culture period, the cells were washed three times with sterile PBS, and 0.1% (v/v) Triton X-100 was added to lyse the cells. The lysate was collected and serially diluted, then plated onto LB agar plates. After overnight incubation at 37 °C, the surviving intracellular bacteria were counted and recorded as CFU_12h_. The CFU_0_ values measured in the previous bacterial phagocytosis experiments for each treatment group served as the initial internalized bacterial load for the corresponding group. These values, together with the CFU_12h_ values measured in the intracellular killing experiments, were used to calculate the relative intracellular killing efficiency. The intracellular bacterial survival rate and the relative intracellular bacterial killing efficiency were calculated using the following formulas, respectively: Intracellular bacterial survival rate (%) = CFU_12h_/CFU_0_ × 100%; relative intracellular bacterial killing efficiency (%) = [1 − CFU_12h_/CFU_0_] × 100%. All experiments were performed in five independent replicates.

### 5.13 IF staining

#### 5.13.1 *In vitro* IF staining of BMDMs

Bone marrow-derived macrophages (BMDMs) were seeded into culture plates containing coverslips and subjected to inflammatory stimulation induced by inactivated *S. aureus*; they were then treated with the corresponding materials according to the experimental groups. After treatment, the cells were fixed with 4% paraformaldehyde, permeabilized with Triton X-100, and blocked with goat serum to prevent nonspecific binding. Subsequently, anti-CD86 and anti-CD206 primary antibodies were added, and the samples were incubated overnight at 4 °C. The following day, after washing with PBS, the cells were incubated with the corresponding fluorescently labeled secondary antibodies in the dark, and the nuclei were counterstained with DAPI. Images were acquired using a fluorescence microscope, with consistent microscope settings and exposure parameters maintained across all groups. ImageJ software was used to quantify the relative fluorescence intensities of CD86 and CD206.

#### 5.13.2 IF staining of tibial tissue from infected mice

At the end of the experiment, the infected tibiae were harvested, fixed in 4% paraformaldehyde, and decalcified using EDTA solution. After gradient dehydration and paraffin embedding, consecutive 5-μm-thick sections were prepared. After deparaffinization and rehydration, the sections underwent antigen retrieval, followed by blocking of nonspecific binding sites. The sections were incubated overnight at 4 °C with primary antibodies against iNOS (HY-P86080, MedChemExpress) and TGF-β1 (HY-P86743, MedChemExpress), respectively. The following day, after washing with PBS, the sections were incubated with the corresponding fluorescently labeled secondary antibodies in the dark, and the nuclei were counterstained with DAPI. Images for each group were acquired using a fluorescence microscope under identical magnification, exposure time, and image acquisition parameters. ImageJ software was used to quantify the relative fluorescence intensities of iNOS and TGF-β1. Consistent parameters were used for all image acquisition and quantitative analyses. The tissue IF signals of iNOS and TGF-β1 were used to assess changes in the local pro-inflammatory and repair-related microenvironment of the infected tibiae.

### 5.14 RT-qPCR

Total RNA was extracted from cells using TRIzol reagent according to the manufacturer’s protocol. RNA concentration and purity were measured before reverse transcription. cDNA was synthesized using a reverse transcription kit. Quantitative PCR was performed using SYBR Green qPCR master mix on a real-time PCR system. GAPDH was used as the internal reference gene. Relative gene expression was calculated using the 2^−ΔΔCT^ method. The detected genes included *Bmp2*, *Smad1*, *IL-6*, and *IL-10*. The primer sequences used are shown in Supplementary Table S1.

### 5.15 Western blot analysis

Under infection-induced stress conditions, MC3T3-E1 cells were treated with different formulations. After treatment, the cells were washed with pre-chilled PBS, and total protein was extracted using RIPA lysis buffer containing protease and phosphatase inhibitors. Protein concentrations in each sample were determined using a BCA protein assay kit. Equal amounts of protein were separated by SDS-PAGE and transferred onto PVDF membranes. After blocking with 5% non-fat dry milk at room temperature for 1 h, the membranes were incubated overnight at 4 °C with primary antibodies against p-Smad1/5/9 (AF8313, Affinity), total Smad1/5/9 (ab300165, Abcam), and GAPDH (60004-1-Ig, Proteintech). The following day, after washing, the membranes were incubated with the corresponding HRP-conjugated secondary antibodies at room temperature for 1 h. Protein bands were visualized using an enhanced chemiluminescence (ECL) system, with GAPDH serving as the loading control.

The Western blot experiment was performed using three independent biological replicates, each derived from independently cultured and treated cell samples. ImageJ software was used to measure the band intensities of p-Smad1/5/9 and total Smad1/5/9. After subtraction of the corresponding background signals, the densitometric ratio of p-Smad1/5/9 to total Smad1/5/9 was calculated and used as a quantitative indicator of Smad1/5/9 phosphorylation. Quantitative results were expressed as the mean ± standard deviation. Differences among groups were analyzed using one-way ANOVA followed by Tukey’s multiple-comparisons test. P < 0.05 was considered statistically significant.

### 5.16 ELISA

The levels of inflammatory cytokines in cell culture supernatants were measured using ELISA kits according to the manufacturers’ instructions. Briefly, supernatants were collected after treatment and centrifuged to remove cell debris. Standards and samples were added to ELISA plates, followed by incubation with detection antibodies and substrate solution. Absorbance was measured using a microplate reader. The concentrations of IL-1β and TNF-α were calculated according to the standard curves.

### 5.17 Establishment of the chronic osteomyelitis mouse model

The mouse tibial osteomyelitis model was established based on previously reported models of long-bone infection, with slight modifications (Lu et al., 2023; Chu et al., 2024). After anesthetizing the mouse, the right hind limb was shaved and disinfected. A small incision was made on the medial aspect of the proximal tibia, and the proximal tibial bone surface was exposed through layer-by-layer dissection. A cortical entry hole was created in the proximal tibia using a 27-G needle; the needle was then advanced along the longitudinal axis of the tibia into the medullary cavity, taking care to avoid penetrating the opposite cortex. Using a microsyringe, 3 μL of a *S. aureus* suspension with a concentration of 2 × 10^8^ CFU/mL was slowly injected into the medullary cavity of the tibia at a rate of 1 μL/10 s, resulting in an inoculum of 6 × 10^5^ CFU per mouse. Since the injection volume was only 3 μL, no bone marrow aspiration or additional decompression was performed during the modeling process. After completion of the injection, the needle was left in place for 30 s and then slowly withdrawn to minimize backflow of the bacterial suspension along the needle tract. The cortical entry hole was immediately sealed with bone wax, and the incision was closed layer by layer using 5–0 sutures. Small-volume inoculation, slow injection, leaving the needle in place after injection, and sealing the site with bone wax were collectively employed to reduce the risk of bacterial suspension backflow and extravasation into surrounding soft tissues.

On day 7 post-inoculation, mice were examined macroscopically for signs such as peritibial abscess formation, increased circumference of the affected tibial region, and impaired use of the affected limb. These indicators served only as auxiliary clinical evidence for the formation of tibial infection and animal randomization; they were not independent criteria for determining intramedullary infection or the development of chronic osteomyelitis. Once the formation of an observable tibial infection was confirmed, the mice were randomly assigned to groups and the corresponding treatment was initiated. The validity of the model and the phenotype of chronic bone infection were comprehensively evaluated at the 4-week endpoint by assessing the bacterial load in the bone marrow of the untreated COM group, bacterial staining of bone tissue, histopathological inflammation and bone destruction, as well as changes in bone structure as shown by micro-CT.

This study did not use fluorescently labeled bacterial suspensions or intraoperative imaging methods to directly verify the intramedullary localization of the inoculum in each animal. The intramedullary placement of the inoculum was primarily controlled by the direction and depth of needle insertion and was indirectly supported by subsequent results, including bone marrow bacterial culture, bacterial staining of bone tissue, intramedullary inflammatory responses, and infection-related changes in bone structure. Therefore, minor reflux of the bacterial suspension along the needle tract or extravasation into surrounding soft tissues could not be completely ruled out.

### 5.18 *In vivo* treatment

For treatment intervention, the mice were randomly assigned to three groups: the COM group, the ZIF-67 group, and the POP@ZIF-67 group (n = 3 per group). Mice in the COM group received an equal volume of PBS, while mice in the ZIF-67 and POP@ZIF-67 groups received 3 μL of ZIF-67 or POP@ZIF-67 nanoparticle suspensions (50 μg/mL), respectively. All treatments were administered via local injection into the tibial medullary cavity every 3 days for a duration of 4 weeks.

### 5.19 Bacterial burden analysis

At the experimental endpoint, infected tibias were collected under sterile conditions. Bone marrow contents were flushed out or homogenized in sterile PBS. The samples were serially diluted and plated onto agar plates. After incubation at 37 °C, bacterial colonies were counted. The bacterial burden was expressed as colony-forming units (CFU).

### 5.20 *In vivo* flow cytometry

Spleens were harvested after treatment and mechanically dissociated to prepare single-cell suspensions. Red blood cells were lysed using red blood cell lysis buffer. After washing with PBS, cells were blocked and incubated with antibodies against F4/80 (Fluor@700), CD11b (PE), CD86 (PC5.5) and CD206 (FITC). After staining, cells were washed and resuspended for flow cytometry analysis. The proportions of CD86^+^ and CD206^+^ cells were quantified. Data were analyzed using FlowJo software.

### 5.21 H&E staining

Tibial samples were fixed in 4% paraformaldehyde and decalcified with EDTA solution. After dehydration and paraffin embedding, samples were sectioned into 5 μm slices. Hematoxylin and eosin (H&E) staining was performed to evaluate inflammatory infiltration and tissue structure. Giemsa staining was used to observe bacterial retention in bone tissues. Masson’s trichrome staining was performed to evaluate collagen deposition and matrix remodeling. Stained sections were observed and photographed using a light microscope.

### 5.22 Micro-CT scanning and analysis

After treatment, the operated tibiae were removed from the mice, the surrounding soft tissue was carefully removed, and the tibiae were fixed in 4% paraformaldehyde overnight. Prior to scanning, all samples were secured in sample tubes in the same orientation to ensure that the long axis of the tibia remained parallel to the longitudinal axis of the scanner and to prevent sample displacement during scanning. Scanning was then performed using a high-resolution micro-CT system (SkyScan 1,276, Bruker, Belgium). The scanning parameters were set as follows: isotropic voxel size of 20 μm, tube voltage of 54 kVp, tube current of 200 μA, single-projection exposure time of 700 ms, rotation step of 0.4°, and a 0.25 mm aluminum filter. Flat-field correction was enabled during scanning; frame averaging and random movement were not applied.

The raw projection data were reconstructed into two-dimensional grayscale tomographic images using NRecon software (version 1.7.4.2, Bruker, Belgium). All samples were reconstructed using the same parameters, including a Hamming filter (α = 0.54, with a cutoff at 100% of the Nyquist frequency), a smoothing parameter of 0, ring artifact correction of 0, and beam hardening correction of 0%. All reconstructed datasets were uniformly resampled to an isotropic voxel size of 20 μm to minimize the influence of differences in the original reconstruction resolution on quantitative bone microstructural measurements.

Subsequently, all samples were reoriented and consistently aligned in the coronal, sagittal, and transverse planes using DataViewer software (Bruker, Belgium), with the proximal tibial growth plate and tibial long axis serving as anatomical landmarks. CTAn software (Bruker, Belgium) was used for region of interest (ROI) delineation, image segmentation, and quantitative analysis of bone microstructure. Using the proximal tibial growth plate as a consistent anatomical reference, the analysis region was defined as slices 34–74 distal to the growth plate, comprising 41 consecutive slices. Based on a voxel size of 20 μm, the ROI began approximately 0.68 mm distal to the growth plate and extended approximately 0.82 mm longitudinally. The same anatomical starting point, ROI length, and contouring criteria were applied to all samples.

In each transverse slice, an irregular anatomical contour was semi-automatically delineated along the endocortical boundary to include the trabecular compartment while excluding the cortical bone and tissues outside the ROI. A uniform global grayscale threshold of 70–255 (8-bit grayscale range) was used to distinguish mineralized bone tissue from non-mineralized tissue; the same threshold and analysis parameters were applied to all samples. The segmentation results were visually inspected by comparing the original grayscale images with the segmented binary images on a slice-by-slice basis to ensure that inappropriate threshold settings did not result in apparent loss or artifactual thickening of the bone structures.

BMD measurements were calibrated against hydroxyapatite calibration phantoms with densities of 0.25 and 0.75 g HA/cm^3^ and expressed in g HA/cm^3^. Bone microstructural parameters were calculated using direct three-dimensional algorithms that did not rely on predefined rod- or plate-shaped structural assumptions, including bone volume fraction (BV/TV), trabecular number (Tb.N), trabecular thickness (Tb.Th), and trabecular separation (Tb.Sp). BV/TV was expressed as a percentage, Tb.N as mm^-1^, and Tb.Th and Tb.Sp as mm. Three-dimensional bone morphology was reconstructed and visualized using CTVol software (Bruker, Belgium); this is for qualitative demonstration purposes only and does not fully correspond to the ROI. All image acquisition, processing, and quantitative analysis procedures were performed in accordance with standardized guidelines for the micro-CT evaluation of rodent bone microstructure (Bouxsein et al., 2010).

### 5.23 IHC staining

OCN expression in tibial sections was detected by immunohistochemical staining. Briefly, paraffin sections were dewaxed, rehydrated, and subjected to antigen retrieval. After blocking endogenous peroxidase activity and non-specific binding, sections were incubated with the primary antibody against OCN at 4 °C overnight. The sections were then incubated with secondary antibody and visualized using DAB substrate. Nuclei were counterstained with hematoxylin. Images were captured using a light microscope, and the OCN-positive area was quantified using ImageJ software.

### 5.24 *In vivo* biosafety evaluation

Major organs, including the heart, liver, spleen, lung, and kidney, were collected after treatment. Samples were fixed in 4% paraformaldehyde, embedded in paraffin, sectioned, and stained with H&E. H&E morphology was observed under a light microscope to evaluate potential systemic toxicity.

### 5.25 Statistical analysis

All data are presented as mean ± standard deviation (SD). Statistical analysis was performed using GraphPad Prism software. Data distribution was assessed using normality tests and homogeneity of variance tests before statistical analysis. Differences between two groups were analyzed using Student’s t-test. Differences among multiple groups were analyzed using one-way analysis of variance (ANOVA) followed by Tukey’s multiple comparisons test. Two-way ANOVA was used for CCK-8 assays involving both treatment groups and time points. A value of p < 0.05 was considered statistically significant, with significance levels denoted as *P < 0.05, **P < 0.01, ***P < 0.001 and ****P < 0.0001.

## Data Availability

The raw data supporting the conclusions of this article will be made available by the authors, without undue reservation.
