# Supplementary material for: Natural polysaccharide-engineered POP@ZIF-67 nanoparticles coordinate antibacterial activity, macrophage polarization, and osteogenesis for chronic osteomyelitis therapy
Source: Front Bioeng Biotechnol. 2026 Oct 7;14:1935416. doi: 10.3389/fbioe.2026.1935416 (PMC13683671; doi:10.3389/fbioe.2026.1935416)
Supplement: Supplementary file 1 [file DataSheet1.pdf]

Supporting Information

# Natural polysaccharide-engineered POP@ZIF-67 nanoparticles coordinate antibacterial activity, macrophage polarization, and osteogenesis for chronic osteomyelitis therapy

Yunchang Zhao[1,2†], Guangting Zhu[1,2†], Mengxue Zhang[1,2†], Yize Wu[1,2], Junwei Su[1,2], Jing Hu[1,2*], Chao Jian[1,2*], Ying Yuan[1,2*]

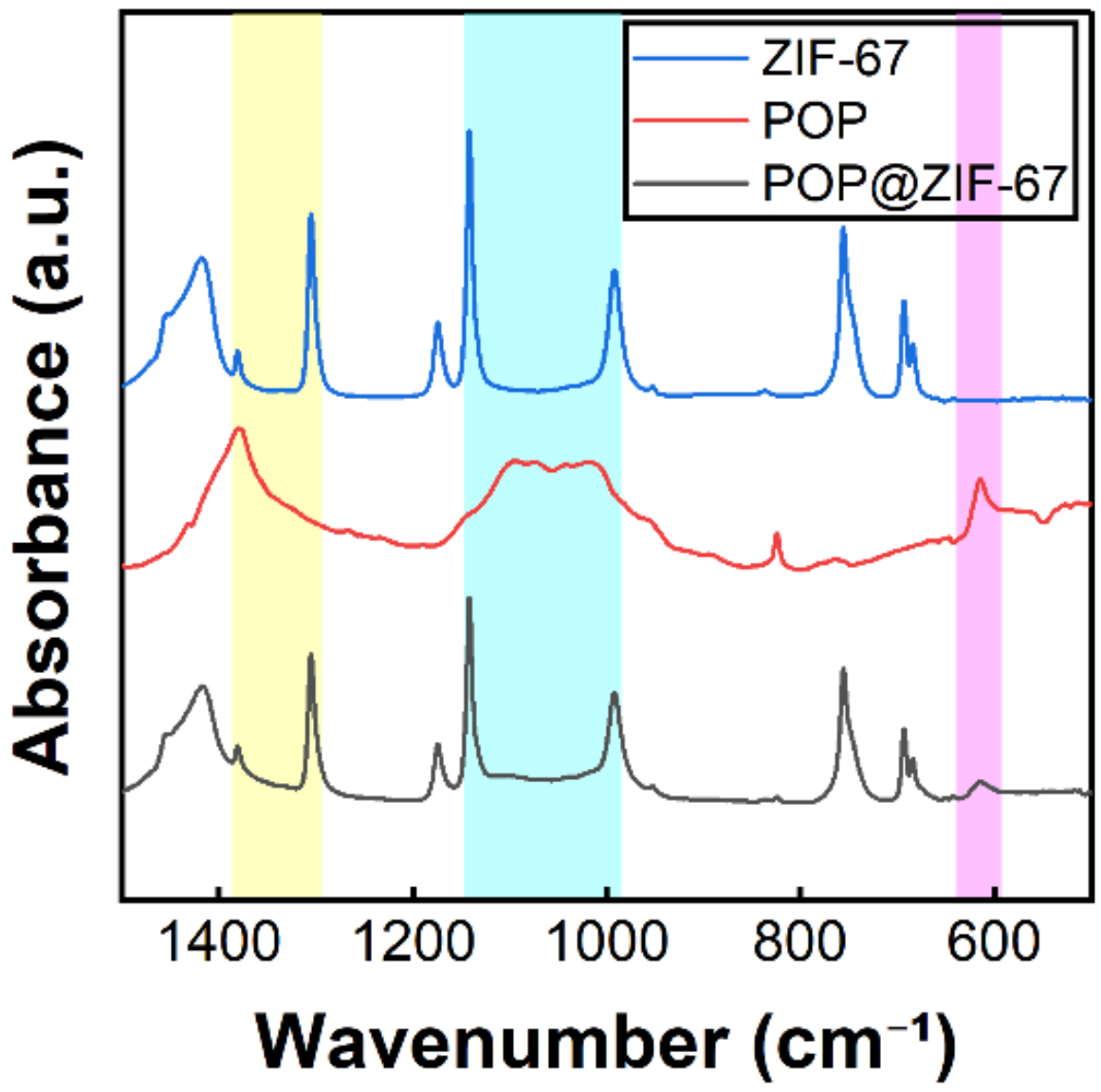


**Figure S1.** Enlarged FTIR spectra of ZIF-67, POP, and POP@ZIF-67 in the wavenumber range of 800–1400 $cm^{-1}$.

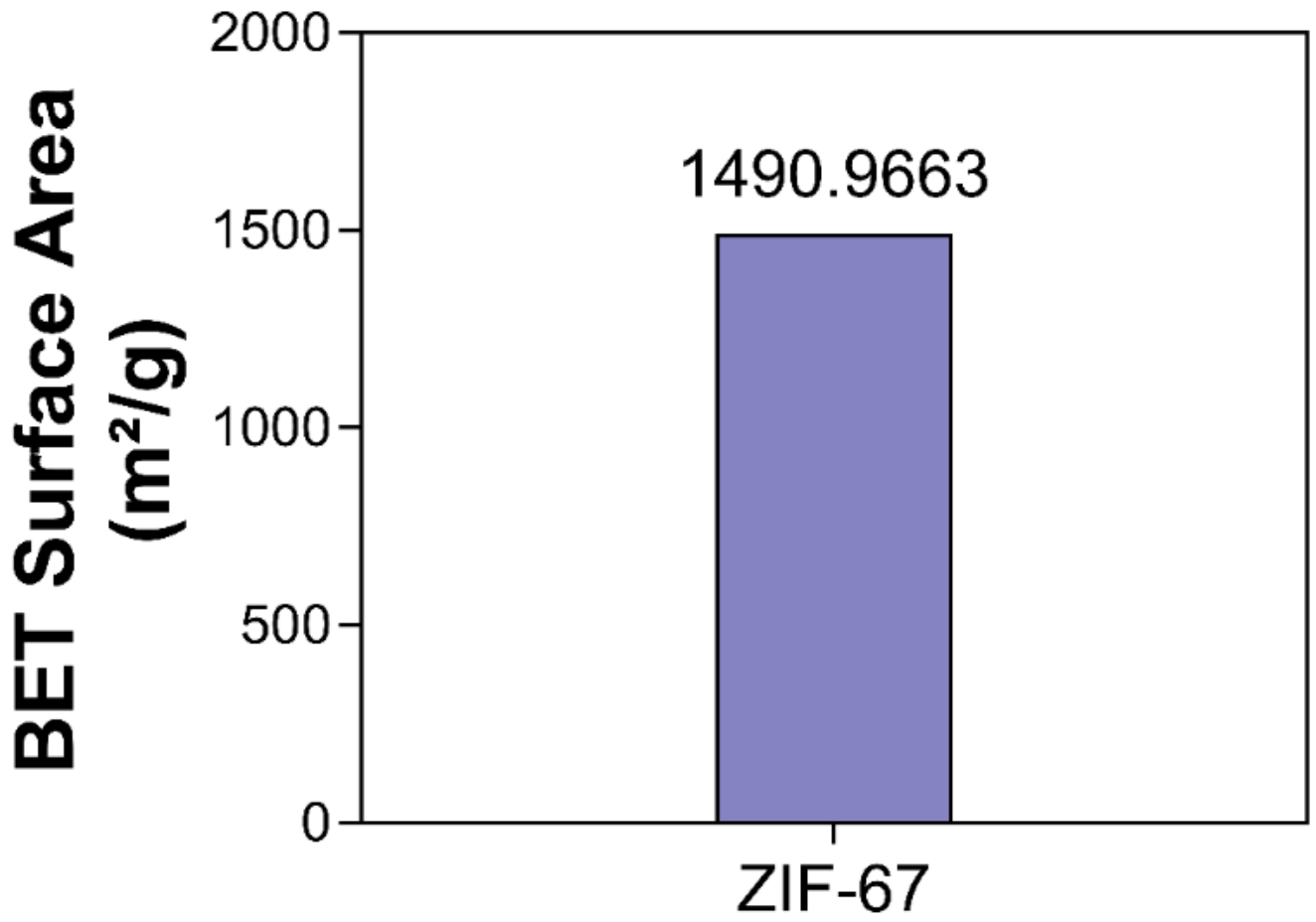


**Figure S2.** BET surface area analysis of ZIF-67.

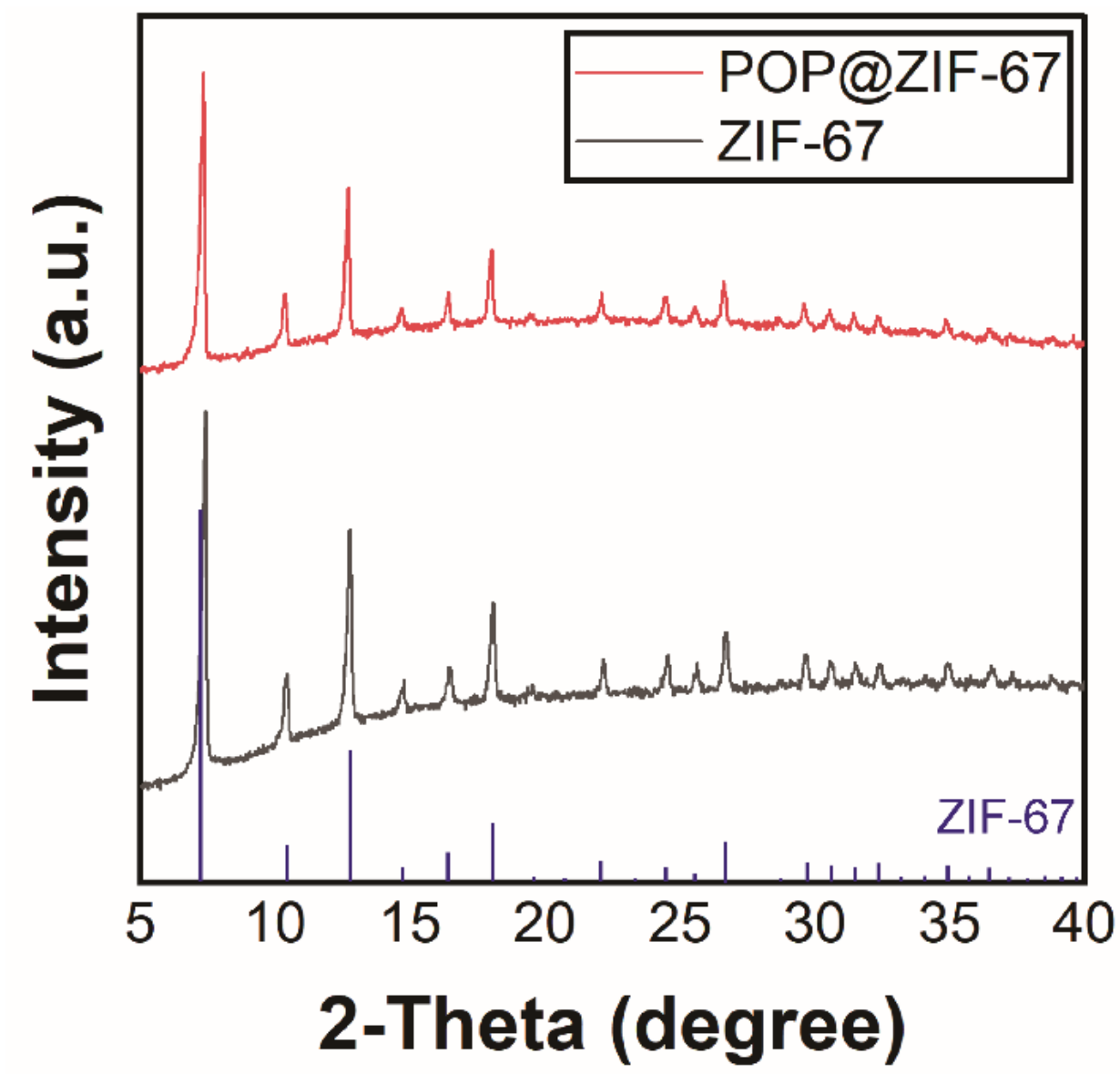


**Figure S3.** XRD patterns of ZIF-67 and POP@ZIF-67.

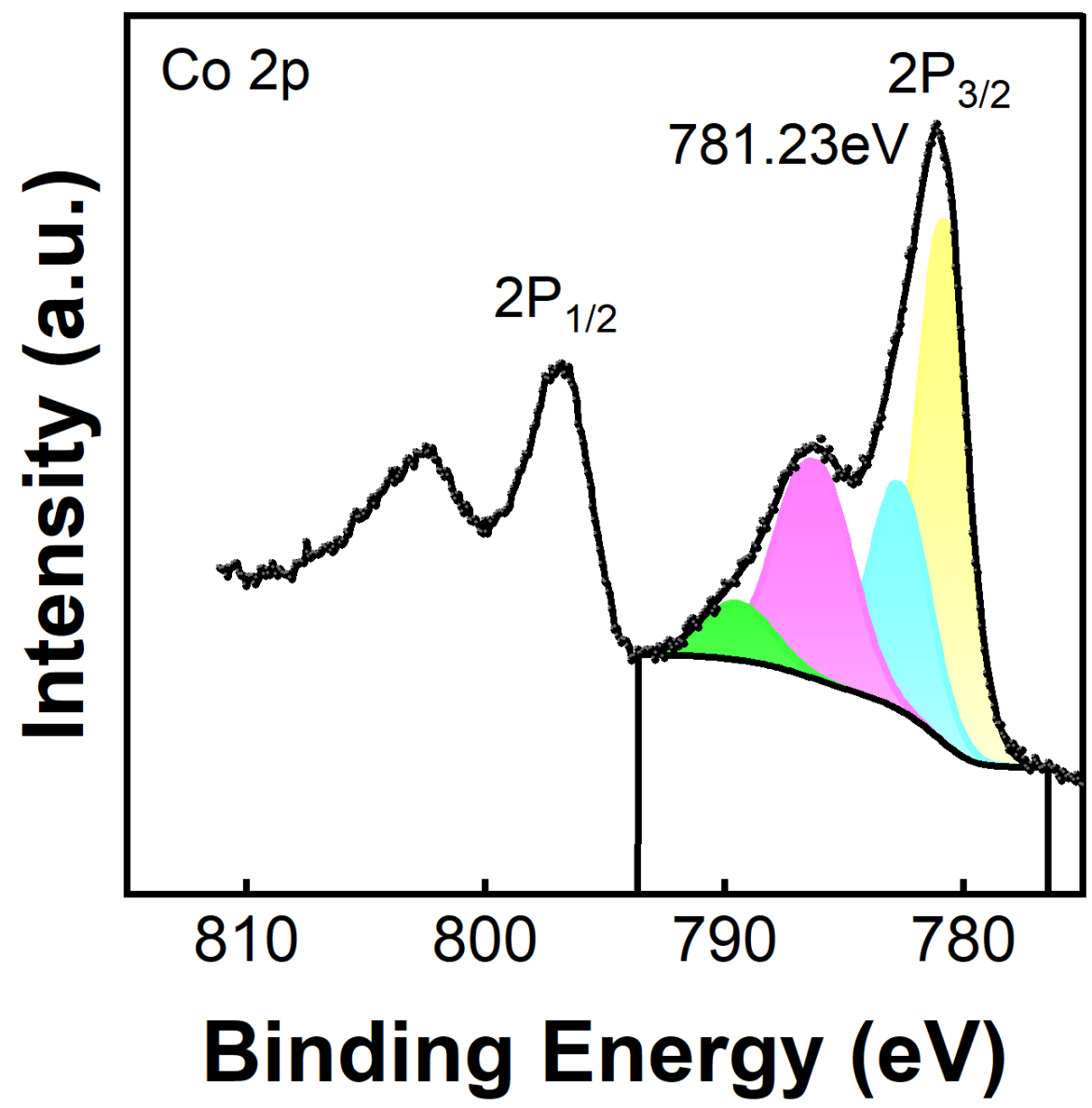


**Figure S4.** XPS spectrum of Co 2p in POP@ZIF-67.

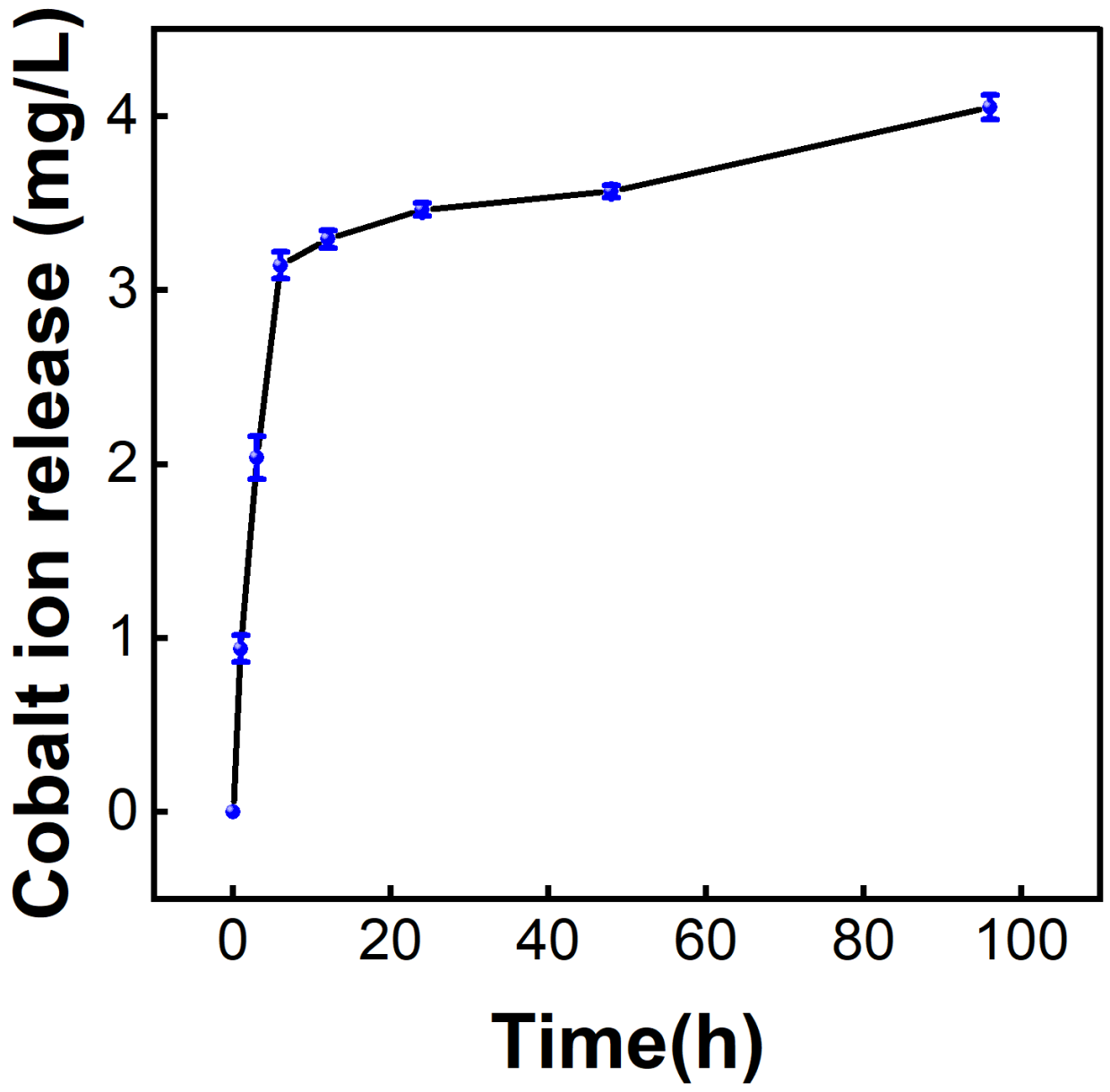


**Figure S5.** Cobalt ion release profile of POP@ZIF-67.

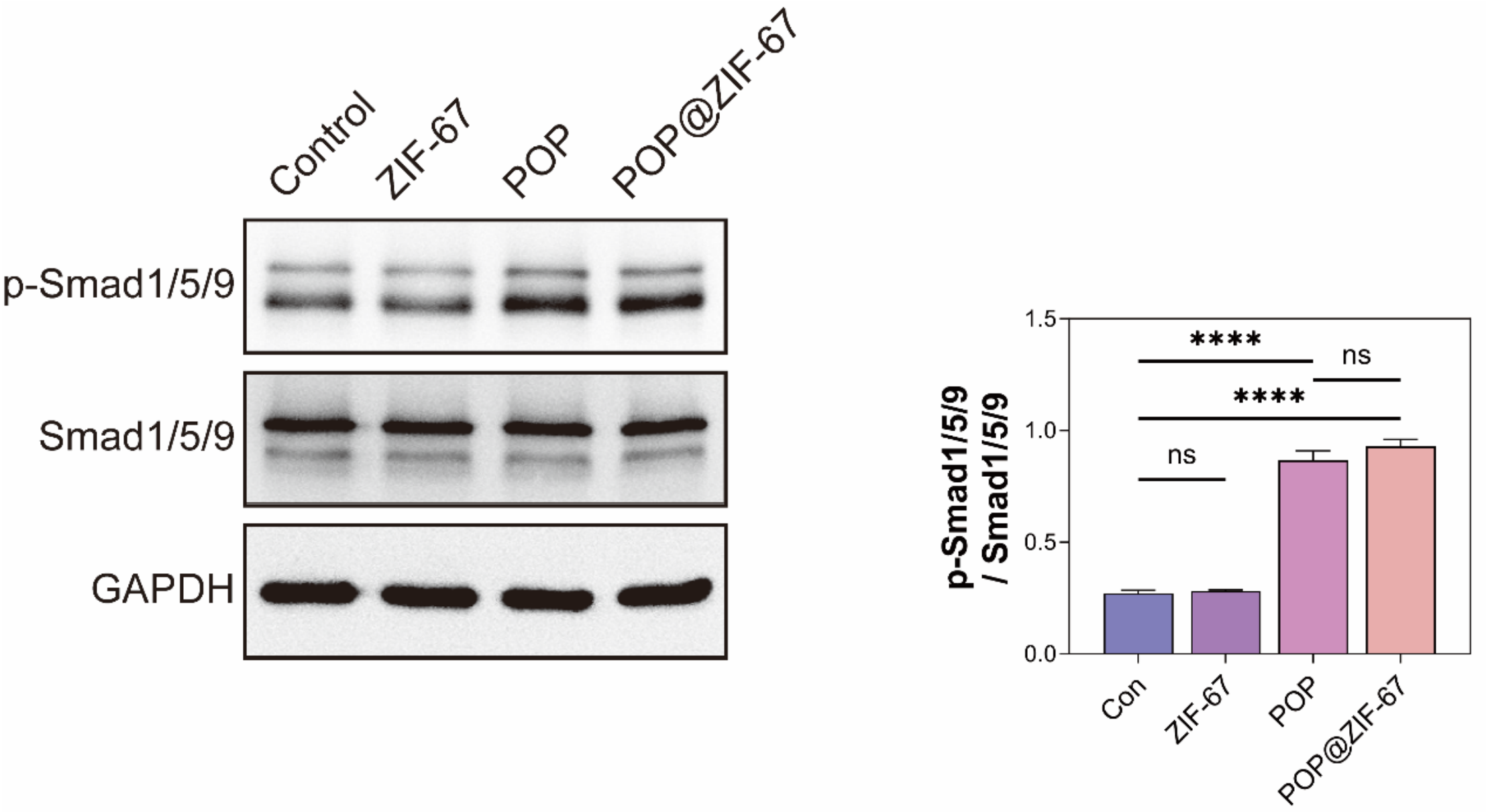


**Figure S6.** Western blot analysis and quantification of BMP/Smad signaling-related proteins in MC3T3-E1 cells after different treatments. Representative Western blot bands showing p-Smad1/5/9 and total Smad1/5/9 expression in MC3T3-E1 cells treated with Control, ZIF-67, POP, and POP@ZIF-67, together with densitometric quantification of the p-Smad1/5/9-to-Smad1/5/9 ratio. GAPDH was used as the loading control. Data are presented as mean ± SD; n = 3 independent biological replicates. Statistical significance was assessed by one-way ANOVA followed by Tukey's multiple-comparisons test. ns, not significant; ****$P < 0.0001$.

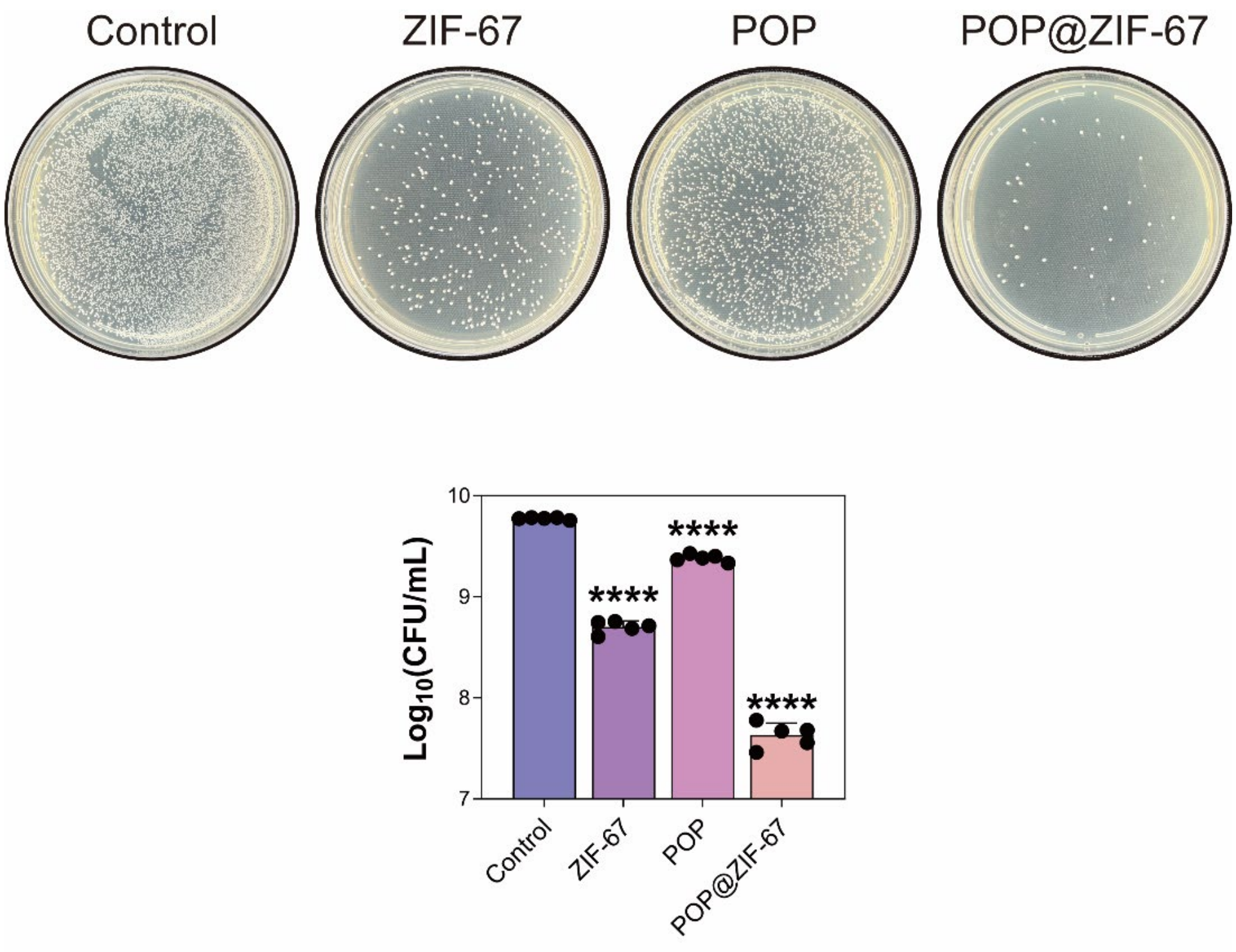


**Figure S7.** Initial internalized bacterial load ($CFU_0$) in RAW264.7 macrophages after different treatments. Representative bacterial colony formation images and quantitative analysis of intracellular bacterial survival after treatment with Control, ZIF-67, POP, and POP@ZIF-67. Data are presented as mean ± SD. n = 5 independent experiments. ****$p < 0.0001$.

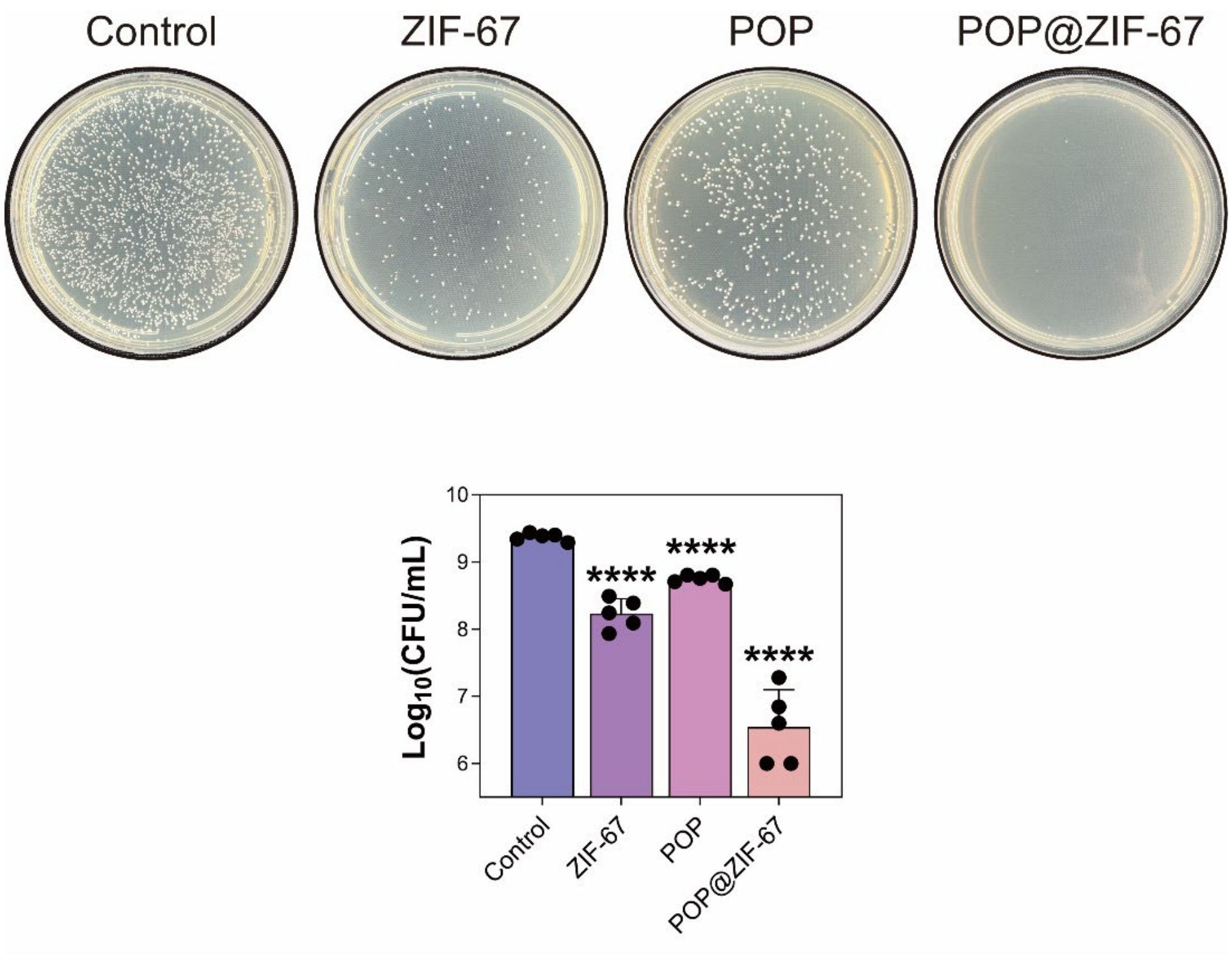


**Figure S8.** Viable intracellular bacterial burden at 12 h after extracellular bacterial elimination. Representative LB agar plates and quantification of viable intracellular

bacteria recovered 12 h after extracellular bacterial elimination. CFU values are expressed as $log_{10}$(CFU/mL). Data are presented as mean ± SD; n = 5 independent experiments. ****p < 0.0001.

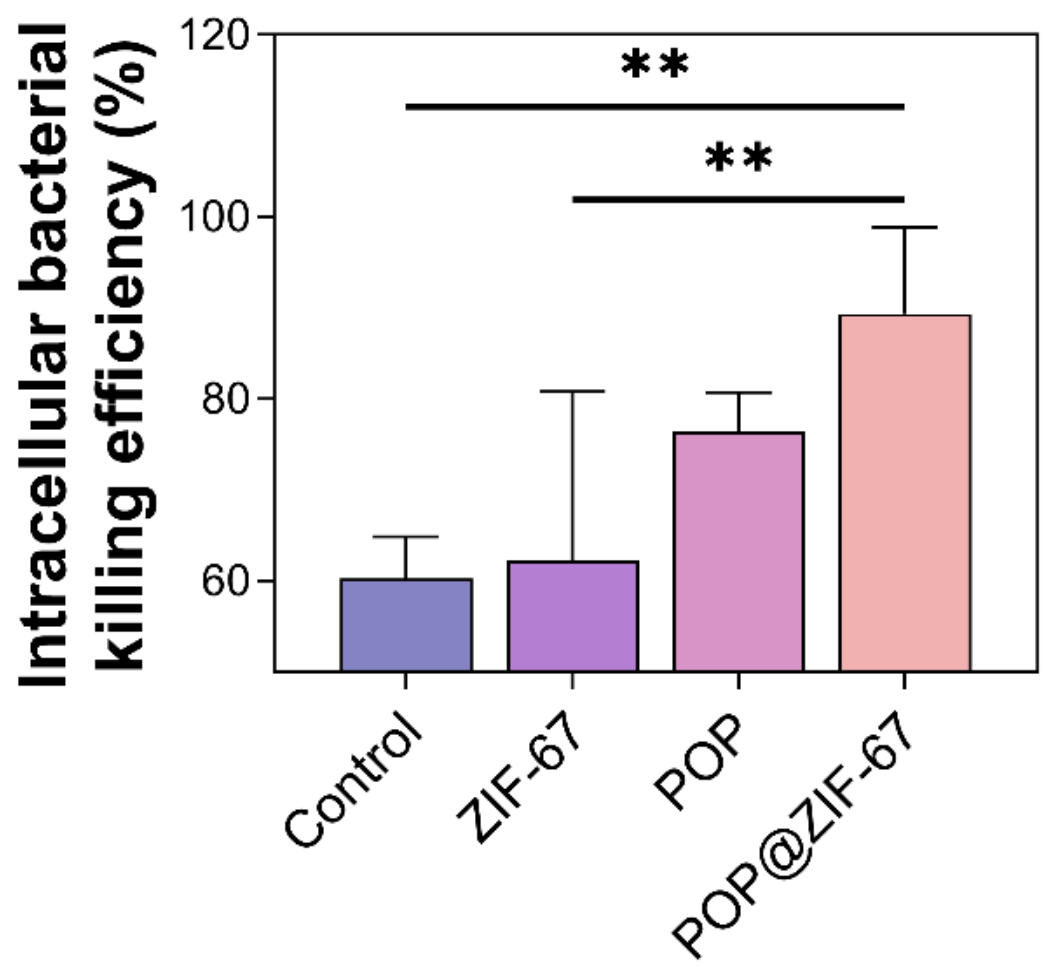


**Figure S9.** Intracellular bacterial killing efficiency of macrophages following different treatments. Killing efficiency was calculated as $[1 - (CFU_{12h}/CFU_0)] \times 100\%$, where $CFU_0$ represents the initial internalized bacterial load and $CFU_{12h}$ represents the surviving intracellular bacterial load after 12 h. Data are presented as mean ± SD; n = 5 independent experiments. Statistical significance was assessed by one-way ANOVA followed by Tukey's multiple-comparisons test; **P < 0.01.

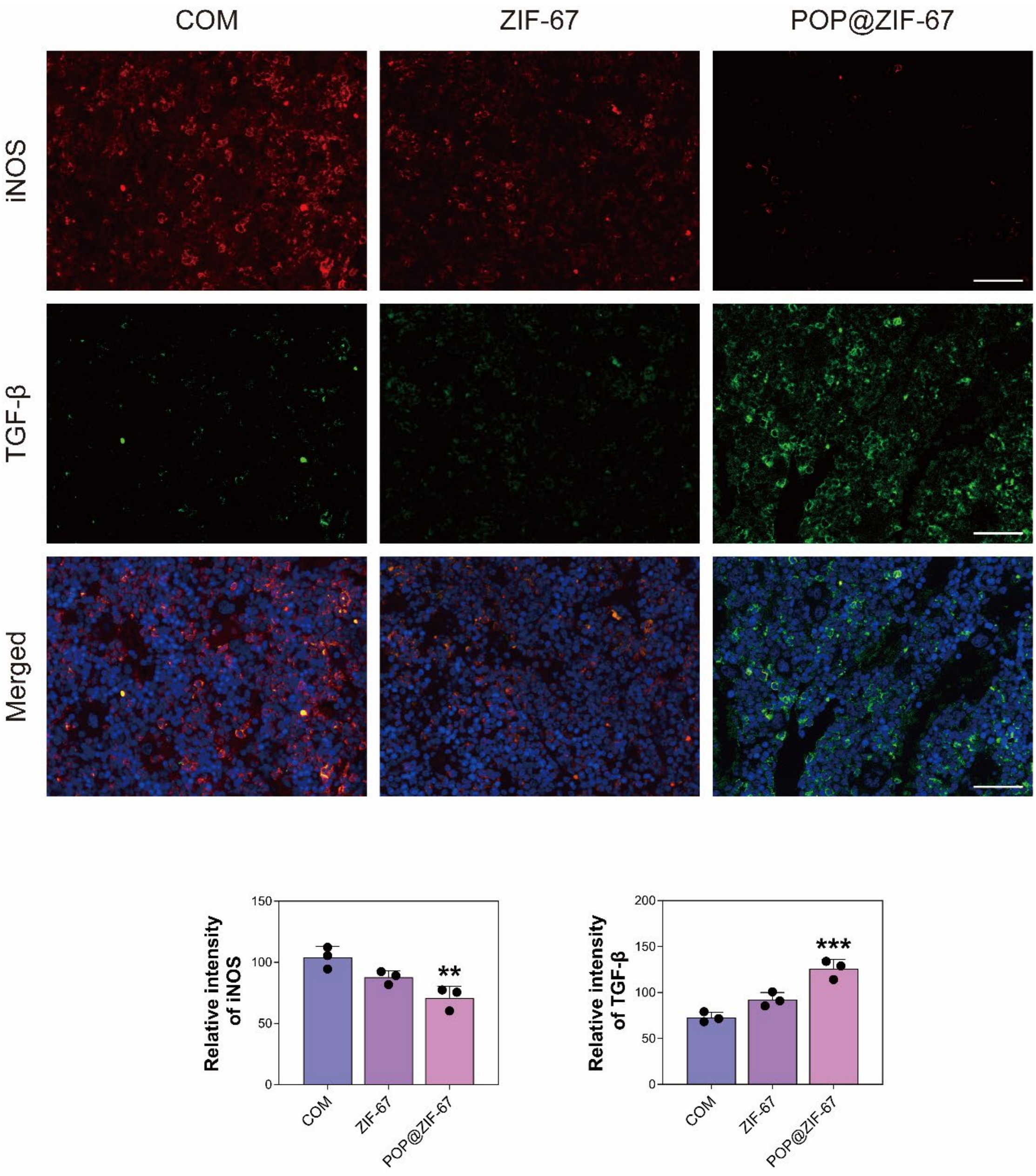


**Figure S10.** Immunofluorescence analysis of inflammatory microenvironment in infected tibial bone marrow after different treatments. Representative immunofluorescence images of iNOS and TGF-β staining in infected tibial bone marrow tissues. Scale bar, 50 µm. Data are presented as mean ± SD; n = 3 independent experiments. Statistical significance was assessed by one-way ANOVA followed by Tukey's multiple-comparisons test; $**P < 0.01$, $***P < 0.001$.

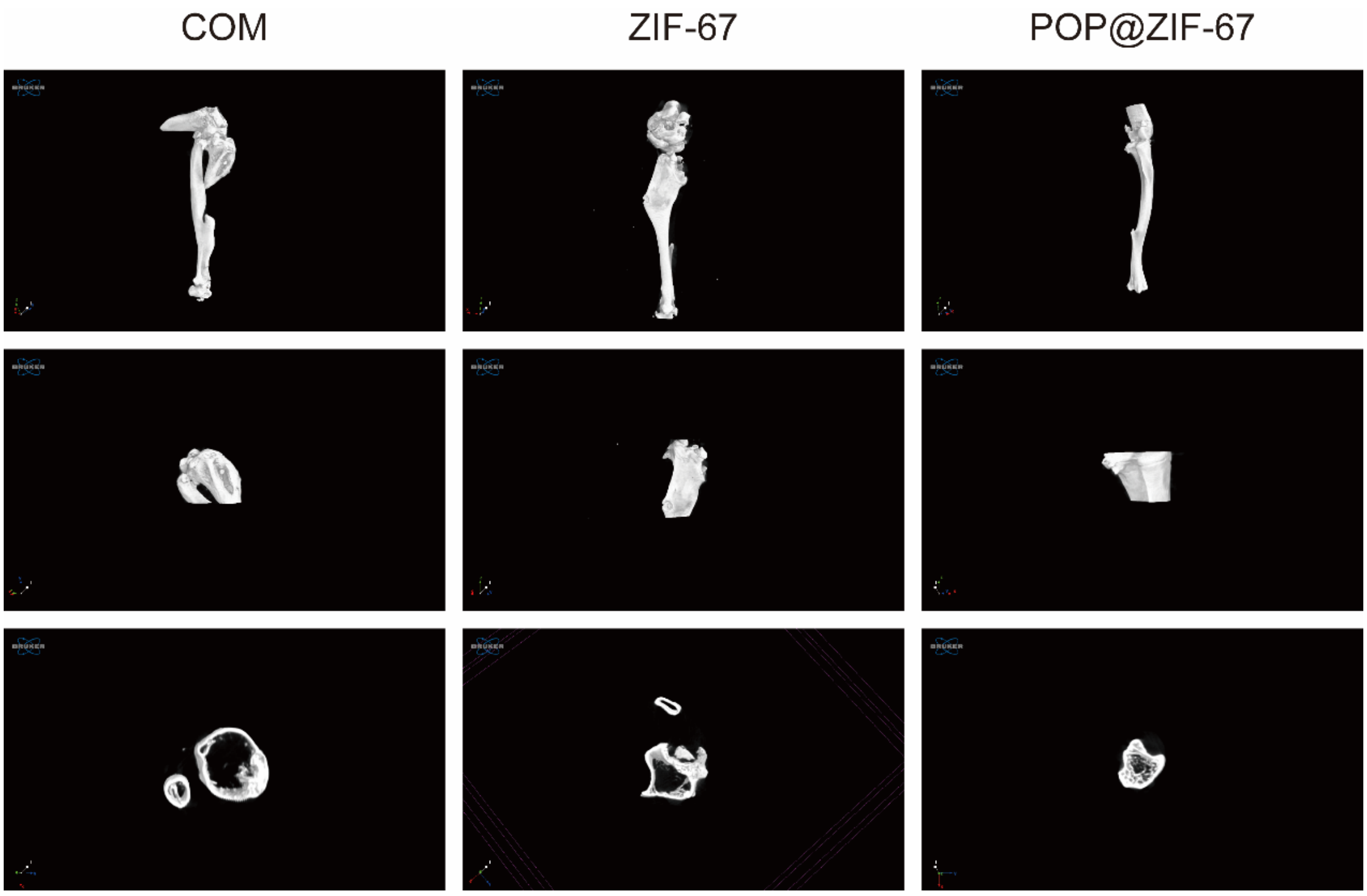


**Figure S11.** Standardized three-dimensional reconstruction of tibial bone structures after different treatments. Representative 3D micro-CT reconstructions of tibiae from the COM, ZIF-67, and POP@ZIF-67 groups.

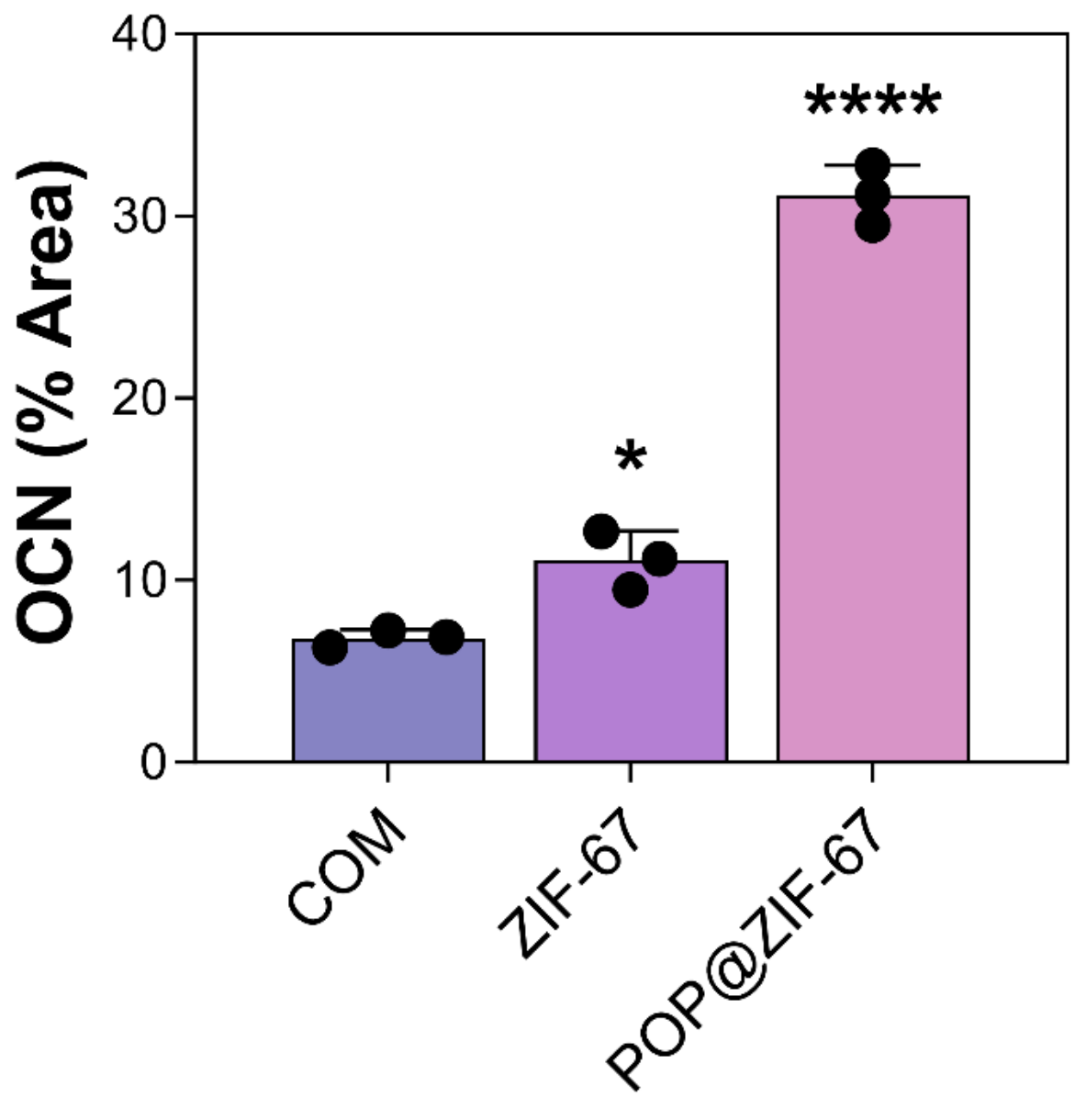


**Figure S12.** Quantification of OCN-positive area. Data are presented as mean ± SD. *p $< 0.05$, ****p $< 0.0001$.

**Table S1.** Primer sequences for RT-qPCR (*in vitro*).

| Primer | Forward | Reverse |
|---|---|---|
| GAPDH | CCTCGTCCCGTAGACAAAATG | TGAGGTCAATGAAGGGGTCGT |

| | | |
|---|---|---|
| BMP2 | AGATCTGTACCGCAGGCACT | CCTCCACGGCTTCTTCGTG |
| Smad1 | CAGGCAGTTGCTTACGAGGA | TCCGGTTAACGTTGGAGAGC |
